# Gut microbiome-derived phenyl sulfate contributes to albuminuria in diabetic kidney disease

**DOI:** 10.1038/s41467-019-09735-4

**Published:** 2019-04-23

**Authors:** Koichi Kikuchi, Daisuke Saigusa, Yoshitomi Kanemitsu, Yotaro Matsumoto, Paxton Thanai, Naoto Suzuki, Koki Mise, Hiroaki Yamaguchi, Tomohiro Nakamura, Kei Asaji, Chikahisa Mukawa, Hiroki Tsukamoto, Toshihiro Sato, Yoshitsugu Oikawa, Tomoyuki Iwasaki, Yuji Oe, Tomoya Tsukimi, Noriko N. Fukuda, Hsin-Jung HO, Fumika Nanto-Hara, Jiro Ogura, Ritsumi Saito, Shizuko Nagao, Yusuke Ohsaki, Satoshi Shimada, Takehiro Suzuki, Takafumi Toyohara, Eikan Mishima, Hisato Shima, Yasutoshi Akiyama, Yukako Akiyama, Mariko Ichijo, Tetsuro Matsuhashi, Akihiro Matsuo, Yoshiaki Ogata, Ching-Chin Yang, Chitose Suzuki, Matthew C. Breeggemann, Jurgen Heymann, Miho Shimizu, Susumu Ogawa, Nobuyuki Takahashi, Takashi Suzuki, Yuji Owada, Shigeo Kure, Nariyasu Mano, Tomoyoshi Soga, Takashi Wada, Jeffrey B. Kopp, Shinji Fukuda, Atsushi Hozawa, Masayuki Yamamoto, Sadayoshi Ito, Jun Wada, Yoshihisa Tomioka, Takaaki Abe

**Affiliations:** 1 Division of Nephrology, Endocrinology and Vascular Medicine, Tohoku University Graduate School of Medicine, Sendai, 980-8574 Japan; 2 Department of Clinical Biology and Hormonal Regulation, Tohoku University Graduate School of Medicine, Sendai, 980-8574 Japan; 3 Department of Integrative Genomics, Tohoku Medical Megabank Organization, Tohoku University, Sendai, 980-8573 Japan; 4 Laboratory of Oncology, Pharmacy Practice and Sciences, Graduate School of Pharmaceutical Sciences, Sendai, 980-8578 Japan; 5 Waters Corporation, Tokyo, 140-0001 Japan; 6 Department of Nephrology, Rheumatology, Endocrinology and Metabolism, Okayama University Graduate School of Medicine, Dentistry and Pharmaceutical Sciences, Okayama, 700-8558 Japan; 7 Department of Pharmaceutical Sciences, Tohoku University Hospital, Sendai, 980-8574 Japan; 8 Department of Preventive Medicine and Epidemiology, Tohoku Medical Megabank Organization, Tohoku University, Sendai, 980-8573 Japan; 9 Department of Pediatrics, Tohoku University Graduate School of Medicine, Sendai, 980-8574 Japan; 10 Division of Clinical Pharmacology and Therapeutics, Tohoku University Graduate School of Pharmaceutical Sciences, Sendai, 980-8578 Japan; 11 Institute for Advanced Biosciences, Keio University, Tsuruoka, 997-0052 Japan; 12 Department of Medical Science, Tohoku University Graduate School of Biomedical Engineering, Sendai, 980-8574 Japan; 13 Education and Research Center of Animal Models for Human Diseases, Fujita Health University, Toyoake, Aichi 470-1192 Japan; 14 Kidney Diseases Branch, NIDDK, NIH, Bethesda, MD 20892-1268 USA; 15 Department of Nephrology and Laboratory Medicine, Kanazawa University, Kanazawa, 920-8641 Japan; 16 Department of Pathology and Histotechnology, Tohoku University Graduate School of Medicine, Sendai, 980-8574 Japan; 17 Department of Organ Anatomy, Tohoku University Graduate School of Medicine, Sendai, 980-8574 Japan; 18 Intestinal Microbiota Project, Kanagawa Institute of Industrial Science and Technology, Kawasaki, 210-0821 Japan; 19 Transborder Medical Research Center, University of Tsukuba, Tsukuba, 305-8577 Japan; 20 PRESTO, Japan Science and Technology Agency, Kawaguchi, 332-0012 Japan

**Keywords:** Microbiome, Metabolomics, Diabetic nephropathy

## Abstract

Diabetic kidney disease is a major cause of renal failure that urgently necessitates a breakthrough in disease management. Here we show using untargeted metabolomics that levels of phenyl sulfate, a gut microbiota-derived metabolite, increase with the progression of diabetes in rats overexpressing human uremic toxin transporter SLCO4C1 in the kidney, and are decreased in rats with limited proteinuria. In experimental models of diabetes, phenyl sulfate administration induces albuminuria and podocyte damage. In a diabetic patient cohort, phenyl sulfate levels significantly correlate with basal and predicted 2-year progression of albuminuria in patients with microalbuminuria. Inhibition of tyrosine phenol-lyase, a bacterial enzyme responsible for the synthesis of phenol from dietary tyrosine before it is metabolized into phenyl sulfate in the liver, reduces albuminuria in diabetic mice. Together, our results suggest that phenyl sulfate contributes to albuminuria and could be used as a disease marker and future therapeutic target in diabetic kidney disease.

## Abstract (web summary)

Diabetes is a major cause of kidney disease. Here Kikuchi et al. show that phenol sulfate, a gut microbiota-derived metabolite, is increased in diabetic kidney disease and contributes to the pathology by promoting kidney injury, suggesting phenyl sulfate could be used a marker and therapeutic target for the treatment of diabetic kidney disease.

## Introduction

Diabetic kidney disease (DKD) occurs in approximately 20–30% of all diabetic subjects and represents a major cause of end-stage renal disease (ESRD), cardiovascular events and death^1,2^. It is difficult to identify type 2 diabetes patients who are at risk of developing progressive DKD based only on measurements of glomerular filtration rate (GFR) and albuminuria^3^. Specific biomarkers are needed for identifying individuals during the early stages of diabetes who are at high risk of complications^2^.

The major function of the kidney is the filtration or transport of various circulating endogenous and exogenous compounds into the urine^4^. Type 2 diabetes causes significant changes in an array of plasma metabolites, even in the early stages^5^. In the kidney, the organic anion transporting polypeptide (OATP) transporter family contributes to the transport of various organic anions and cations into the urine. In rodent kidney, several *oatp* gene products are present at the proximal tubules, whereas SLCO4C1 is the only OATP in human kidney^4^. The diversity among the oatp family members makes it difficult to extrapolate from experimental studies on rodents to humans. To overcome this issue, we generated transgenic rats overexpressing human SLCO4C1 in the proximal tubule^6^. These rats are an excellent model for evaluating the human kidney-specific elimination for metabolites and uremic toxins. We previously clarified that the excretion of uremic toxins in the SLCO4C1 transgenic rat model reduced hypertension, cardiomegaly, and inflammation in the setting of renal failure^6^.

Here, using this model, we characterize metabolites that are increased in diabetic wild-type rats (WT-DM), but reduced in diabetic SLCO4C1 transgenic rats (Tg-DM). We find that levels of phenyl sulfate (PS), a gut microbiota-derived metabolite, significantly correlate with basal and predicted 2-year progression of albuminuria in patients with microalbuminuria. Inhibition of PS production reduces albuminuria in diabetic mice. Together, our results suggest that PS is not only an early diagnosis marker, but also a modifiable cause and therefore a target for the treatment of DKD.

## Results

### Reduction of PS reduced proteinuria in a rat diabetes model

**Fig. 1:**
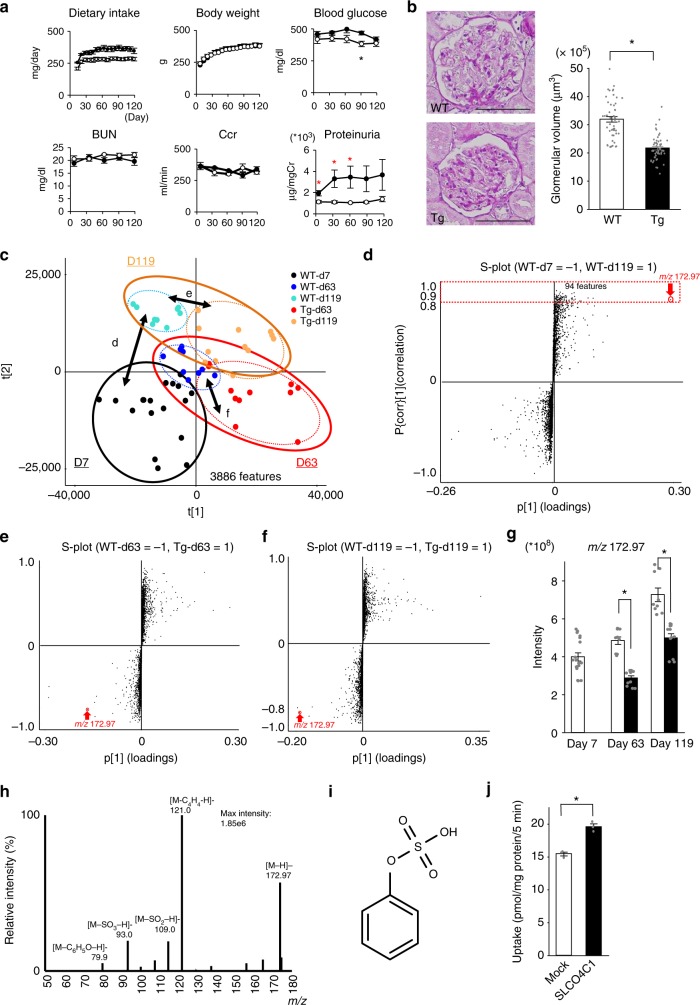
Diabetic SLCO4C1-Tg rats showed reduced proteinuria. **a** Body weight, blood glucose, blood urea nitrogen (BUN), and creatinine clearance (Ccr) in diabetic SLCO4C1-Tg rats (white circles, *n* = 5) and WT rats (black circles, *n* = 6). **b** Representative PAS (left) stained sections of WT and SLCO4C1-Tg rat glomeruli. Bars = 100 μm. *n* = 50 in each group. Data are mean ± SEM. *p < 0.05 vs. WT. Student *t* test. **c** Variation in the five groups in the PLS-DA scores plot using the chemical features detected in plasma. Sample conditions are represented by color coded circles: WT-d7 (black), WT-d63 (blue), WT-d119 (cyan), Tg-d63 (red), and Tg-d119 (orange). Groups D7, D63, and D119 are surrounded by black, red, and orange solid rings, respectively. Groups WT-d63, WT-d119, Tg-d63, and Tg-d119 are surrounded by blue, cyan, red, and orange dotted rings, respectively. **d** *S*-plot analyses of orthogonal partial least square-discriminant analysis (OPLS-DA) of WT rats on days 7 and 119. The selected 94 features (surrounded by a dotted red rectangle) were significantly greater on day 119 than on day 7. **e** *S*-plot analyses of OPLS-DA of WT and Tg rats on day 63. **f** *S*-plot analyses of OPLS-DA of WT and Tg rats on day 119. **g** Changes in the concentration of the selected feature *m/z* 172.97 with the progression of diabetes. Wild-type rats (white column, *n* = 6); SLCO4C1-Tg rats (black column, *n* = 5). **h** Fragment ion mass spectrum of *m/z* 172.97. The main detected precursor and fragment ion was *m/z* 172.97. The product ions found in the corresponding extracted MS^E^ high-energy spectrum at *m/z* 79.9, *m/z* 93.0, *m/z* 109.0, and *m/z* 121.0 were hypothesized to be [M-C_6_H_5_O-H]-, [M-SO_3_-H]-, [M-SO_2_-H]-, and [M-C_4_H_4_-H]-, respectively. **i** Chemical structure of PS. **j** SLCO4C1-mediated PS uptake by SLCO4C1/MDCKII cells (*n* = 3). Data are mean ± SEM. *p < 0.05 vs. control according to Student *t* test (**a**, **b**, **g**, **j**). Source data are provided as a Source Data file and untargeted metabolome data are provided as a Supplementary Data 1

To identify metabolites linked to diabetic conditions, streptozotocin (STZ)-induced diabetes was induced in SLCO4C1-Tg rats on day 0. On day 7, bloods were collected and rats with blood glucose levels greater than 300 mg/dl were selected for further analysis. Dietary intake and body weights were similar in WT-DM and SLCO4C1-Tg DM rats (Fig. 1). Blood glucose levels increased following STZ (~500 mg/dl) and, during the experimental period, blood glucose levels were similar in both groups, except on day 90. The WT-DM and Tg-DM groups showed no differences in renal function. However, proteinuria in the Tg-DM group was significantly lower than in the WT-DM group on days 7, 30, and 63, but showed a non-significant trend on days 90 and 119 (Fig. 1a). Histological analysis showed that glomerulomegaly was present in the WT-DM group, but reduced in the Tg-DM group (Fig. 1b). No significant change was observed in the preserved tubular and fibrotic areas between WT-DM and Tg-DM rats (Supplementary Fig. 1). These data suggest that overexpression of SLCO4C1 in diabetic kidney decreases proteinuria without major histological changes, except in glomeruli at the light microscopic level.

### Identification of PS as a pathogen-derived metabolite in DKD

Since SLCO4C1 is a transporter that eliminates metabolites into urine, we concluded that excretion of a SLCO4C1-specific substrate into urine caused a reduction in proteinuria. Initially, we performed untargeted metabolome profiling to screen for major molecular differences using ultra high-performance liquid chromatography (UHPLC) coupled to quadrupole time-of-flight mass spectrometry (UHPLC-QTOF/MS). Over 4173 features were detected from the 5 groups: WT day 7 (WT-d7), WT day 63 (WT-d63), WT day 119 (WT-d119), SLCO4C1-Tg day 63 (Tg-d63), and SLCO4C1-Tg day 119 (Tg-d119). Totally, 17.6% (or 827) of the features with a coefficient of variation (CV) ≥30% were excluded and the remaining 3886 features were selected (Supplementary Data 1). Principle component analysis (PCA) was conducted on all groups (Supplementary Fig. 2a), all WT groups (days 7, 63, and 119, Supplementary Fig. 2b) and all TG groups (days 63 and 119, Supplementary Fig. 2c). Although some broad-based overlapping clustering can be observed, these analyses showed no clear distinction among the groups. This suggested that the largest source of variation is within each group and not among the groups.

To maximize and analyze differences among the five groups, partial least square-discriminant analysis (PLS-DA) was also performed. Figure 1c shows separation of the five groups analyzed. To evaluate the effect of STZ and highlight the most robust markers of interest, we used orthogonal PLS-DA (OPLS-DA). We compared the plasma metabolomes between WT-d7 and WT-d119 rats, a time interval during which diabetic changes progressed (Fig. 1d). The OPLS-DA scores plot of WT-d7 and WT-d119 mice (Supplementary Fig. 2d) showed good fit and high predictability of the model with values for *R*^2^*Y* (which assesses the extent to which the data fit the model) and the quality assessment metric *Q*^2^ of 0.994 and 0.956, respectively. From the *S*-plot of the OPLS-DA^7^, 94 features that were significantly higher in WT-d119 also had a correlation value (*p*(corr)[1]) >0.85 (up to 1.0). These features were further filtered down to components with a low %CV (<15%) (Supplementary Table 1).

We next looked for analytes with high variable importance in projection (VIP) scores, which assess the relative contribution to the OPLS-DA model. These analyses identified one metabolite (*m/z* 172.97, red circle and arrow) that showed a distinctly strong contribution (high p[1]) relative to the other features, with a fold change of 1.9, a distinct VIP score of 5.9 relative to the other components of VIP score <1.5 (Fig. 1d, Supplementary Table 1). Further investigation revealed that it increased with diabetes progression. This increase was significantly reduced in SLCO4C1-Tg rats when compared to WT rats on days 63 and 119. This metabolite also showed a distinctly strong contribution relative to the other components in the *S*-plot of WT-d63 vs. Tg-d63 (Fig. 1e, red arrow) and the *S*-plot of WT-d119 vs. Tg-d119 (Fig. 1f). In summary, this feature was the only one to fulfill the selection criteria of increasing with the progression of diabetes and being reduced in Tg rats, and led to its selection for further investigation. The change in intensity of *m/z* 172.97 is shown in Fig. 1g. The fold changes and associated *p* values for WT-d63 vs. Tg-d63 and for WT-d119 vs. Tg-d119 are listed in Supplementary Table 1.

To identify the feature, the elemental composition was analyzed and predicted to be C_6_H_5_O_4_S ([M-H]^−^), with a theoretical value of *m/z* 172.97. A search of the Chemspider database using the predicted elemental composition C_6_H_5_O_4_S ([M-H]^−^) revealed the following candidates: 2-hydroxybenzenesulfonate, 3-hydroxybenzenesulfonate, 4-hydroxybenzenesulfonate, and PS. We tried to identify these features by matching fragments of the MS/MS spectra obtained from rat plasma. The product ions found in the corresponding extracted MS^E^ high-energy spectrum at *m/z* 79.9, m/z 93.0, m/z 109.0, and m/z 121.0 were hypothesized to be [M-C_6_H_5_O-H]^−^, [M-SO_3_-H]^−^, [M-SO_2_-H]^−^, and [M-C_4_H_4_-H]^−^, respectively (Fig. 1h). Finally, the identity was confirmed using a synthesized chemical standard of PS (Fig. 1i), which provided a similar fragmentation pattern and retention time. The plasma PS levels measured precisely by liquid chromatography (LC)/MS/MS^8^ in WT-DM rats on days 63 and 119 were 4.47 ± 0.43 and 6.48 ± 0.71 μM, respectively, and these levels were decreased in Tg-DM rats (Supplementary Fig. 3). To confirm whether PS is a substrate of SLCO4C1, an uptake study was performed using SLCO4C1-expressing MDCK (SLCO4C1/MDCKII) cells^9^. The uptake of PS into cells was increased in SLCO4C1/MDCKII cells, verifying PS as a substrate for the SLCO4C1 transporter (Fig. 1j). We also examined PS uptake using human proximal tubular HK-2 cells. The uptake of PS by HK-2 cells was inhibited by the SLCO4C1 inhibitor ritonavir (Supplementary Fig. 4). These data demonstrated that PS increased with the progression of diabetes. Urinary protein and PS accumulated in diabetic rats were reduced by the overexpression of SLCO4C1 in the kidney.

### PS-induced albuminuria by podocyte damage in diabetic models

**Fig. 2:**
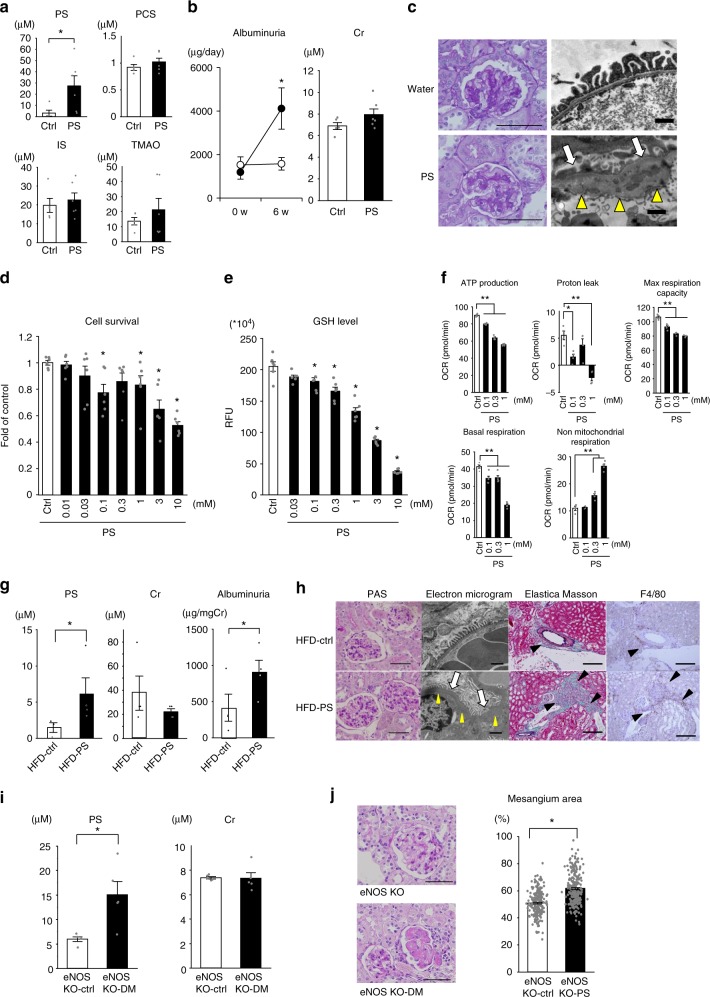
Pathophysiological features of PS in vitro and in vivo. **a** Plasma levels of PS, PCS; IS, and TMAO in 11-week-old db/db mice in control (Ctrl; *n* = 5) or PS (50 mg/kg/day for 6 weeks, *n* = 6). **b** Plasma albumin and Cr levels before and after administering PS for 6 weeks to db/db mice. Control (white circles, *n* = 5) and PS-treated (black circles, *n* = 6) groups. **c** Glomeruli in db/db mouse with or without PS for 6 weeks. Bar, 80 μm (PAS) and 1 μm (EM). **d** Cell toxicity analysis (*n* = 6). **e** Cellular GSH level (*n* = 6). **f** Bioenergetic characterization of cultured human podocytes in terms of oxygen consumption rate (OCR). *n* = 4. **g** PS, albuminuria and Cr levels before and after administering PS for 6 weeks in HFD-KKAy mouse (6 weeks old). For PS, *n* = 3 (HFD-control) and *n* = 4 ((HFD-PS). For Cr and albuminuria, *n* = 4. Wilcoxon (PS) and Student’s *t* test (Cr and albuminuria). **h** Histological images of PAS and electron microscopic analysis of podocytes from control (Ctrl; top row) and PS-treated (PS; bottom row) groups of HFD-KKAy mice. Scale bar = 200 μm for PAS and 1 μm for electron microscopy. The effacement of podocytes (white arrows) and GBM thickness (yellow arrow heads) are shown. The inflammatory area (Elastica Masson) and macrophage infiltration (F4/80 immunostaining) around the vascular area are indicated (black arrow heads). Scale bar, 80 μm. **i** PS levels in eNOS knockout mice with or without diabetes (*n* = 5). **j** Histological examination stained with PAS of eNOS knockout mice with or without diabetes. Scale bar, 50 μm. *p < 0.05 vs. control according to Student *t* (**a, b, d, e, I, j**) or Tukey’s test (**f**). Source data are provided as a Source Data file

We used several models to determine whether PS plays a pathogenic role in the progression of albuminuria and diabetic renal damage. The basal PS level in db/db mice was 3.06 ± 2.63 μM (Fig. 2a, *n* = 5). To accelerate phenotypic changes, we administered PS orally to two types of diabetes models. Oral administration of PS to db/db mice (50 mg/kg) resulted in a rapid rise in plasma PS to 64.53 ± 5.87 μM in 1 h, which then rapidly decreased (*n* = 3, Supplementary Fig. 5). After oral administration of PS to db/db mice for 6 weeks, the plasma level was significantly increased by 5-fold (27.3 ± 9.17 μM, Fig. 2a). There was no change in the biological data between control db/db mice and PS-treated db/db mice (Supplementary Table 2).

Since PS is usually detected in renal failure and recognized as a gut-derived uremic toxin^10^, we measured the plasma level of other gut microbiota-derived uremic toxins (*p*-cresyl sulfate (PCS), indoxyl sulfate (IS), and trimethylamine *N*-oxide (TMAO), Fig. 2a). We found no significant differences in PCS, IS, and TMAO concentrations following PS administration. Albuminuria was increased in db/db mice supplemented with PS (Fig. 2b).

Because podocyte injury causes albuminuria^11^, we focused on the effect of PS on podocytes. Histological examination by light microscopy revealed mesangial expansion, but not significant (Fig. 2c). However, electron microscopy revealed increased the foot processes effacement (Fig. 2c, white arrows) and the glomerular basement membrane thickening (GBM) (Fig. 2c, yellow arrow heads) in PS-treated db/db mice. These data suggest that PS elicits podocyte damage in diabetic mice. In addition, although there was no difference between in the preserved tubular area and fibrotic area at the light microscopic level (Supplementary Fig. 6a), the expression levels of representative inflammatory genes, TNF-α (*Tnfa*) and MCP-1 (*Ccl2*), the fibrotic gene TGF-α_1_ (*Tgfb1*), fibronectin (*Fn1*), and collagen I (*Col1a1*) were increased in PS-treated db/db kidney (Supplementary Fig. 6b). It was reported that db/db mice develop renal fibrosis^12^. Our data further suggest pro-fibrotic and pro-inflammatory effects of PS in the DKD model.

To confirm the toxic effect of PS on podocytes, we employed cell survival analysis using differentiated human urinary podocyte-like epithelial cells (HUPECs)^13^. As shown in Fig. 2d, PS was toxic to differentiated podocytes from 30 μM and significant cell toxicity was seen at concentrations from 100 μM. We also measured the effect of PS on glutathione levels, as it was reported PS decreases glutathione levels, rendering the cells vulnerable to oxidative stress^14^. Glutathione levels in differentiated podocytes exposed to PS (30 μM) decreased and significant at 100 μM (Fig. 2e). This was comparable to reports in CKD (~62 μM)^15^ and in our human DKD study (0–68.1 μM, Table 1).

**Table 1:** Baseline and 2-year after characteristics of the U-CARE study

		Baseline	After 2 years
PS	(μM)	3.3 (0–68.1)	
suPAR	(pg/mL)	460.8 (142.0–2740.2)	
Age	(years)	63.3 ± 12.4	65.3 ± 12.4
Gender	(M/F)	206/156	206/156
DM type	(1/2/others)	40/318/4	
Duration	(years)	14.6 ± 8.8	16.6 ± 8.8
Time to measure	(years)		2.08 ± 0.18
BMI	(kg/m^2)^	25.0 ± 4.4	
SBP	(mmHg)	127.8 ± 15.2	128.6 ± 15.8
DBP	(mmHg)	72.4 ± 10.2	72.1 ± 10.6
BS	(mg/dL)	154.2 ± 56.4	159.5 ± 60.6
HbA1c	(%)	7.2 ± 1.1	7.3 ± 1.1
eGFR	(mL/min/1.73 m^2^)	73.8 (17.1–115.4)	72.6 (13.8–112.4)
ACR	(mg/gCr)	11.0 (1.0–6407.4)	13.1 (1.1–3410.8)
ALT	(IU/L)	22.5 ± 12.5	23.4 ± 14.9
TC	(mg/dL)	175.4 ± 31.6	179.9 ± 32.1
TG	(mg/dL)	132.2 ± 82.6	133.1 ± 79.4
HDL	(mg/dL)	55.3 ± 16.6	57.1 ± 16.7
UA	(mg/dL)	5.3 ± 1.7	5.1 ± 1.4

Baseline and 2-year after characteristics of the U-CARE study (*n* = 362 DKD patients). Mean ± SD was for parametric factors and the median range was for nonparametric factors (eGFR, ACR, PS, and suPAR). In the U-CARE study, eGFR (CKD-EPI) is the estimated eGFR based on the equation of Chronic Kidney Disease Epidemiology Collaboration (CKD-EPI). *PS* phenyl sulfate concentration, *suPAR* soluble urokinase-type plasminogen activator receptor, *duration* duration of diabetic mellitus, *BMI* body mass index, *SBP* systolic blood pressure, *DBP* diastolic blood pressure, *BS* blood glucose level, *HbA1c* NGSP value, *eGFR* estimated glomerular filtration rate (CKD-EPI), *ACR* urinary albumin-to-creatinine ratio, *ALT* alanine aminotransferase, *TC* total cholesterol, *TG* triglyceride, *HDL* high-density lipoprotein cholesterol, *UA* uremic acid.

Since podocyte mitochondrial dysfunction was also reported in the pathogenesis of DKD^16^, we examined the bioenergetic effect of PS on cultured podocytes^17^. By measuring the oxygen consumption rate (OCR), PS (100 μM to 1 mM) exposure to differentiated podocytes significantly decreased mitochondrial basal respiration, ATP production, H^+^ leaking and maximum respiration capacity, suggesting that PS is toxic to mitochondria (Fig. 2f and Supplementary Fig. 7). PS also increased nonmitochondrial respiration, suggesting respiration compensation. Extracellular acidification rate (ECAR) measuring glycolysis did not differ between PS-treated and control differentiated podocytes, further suggesting that glycolysis was not compensated enough.

To confirm the effect of PS in diabetic kidney, we also administered PS (50 mg/kg) to KKAy mice fed on a high-fat diet (HFD), another model of DKD^18^. The plasma PS level was significantly increased to 6.09 ± 3.18 μM (*n* = 4) in PS-treated HFD-KKAy mice compared to non-PS-treated HFD-KKAy mice (1.48 ± 0.85 μM; Fig. 2g, *n* = 3)). The clinical data had not changed (Supplementary Table 3). Oral administration of PS to HFD-KKAy mice for 6 weeks increased albuminuria (Fig. 2g). Electron micrographs revealed podocyte effacement (Fig. 2h, white arrows) and GBM thickening (Fig. 2h, yellow arrow heads) and damage in the PS-treated HFD-KKAy kidney. Elastica Masson and F4/80 staining showed some perivascular fibrotic area in the PS-treated group (Fig. 2h, black arrow heads). These results further suggest that the manifested podocyte damage and perivascular inflammation were elicited by PS, which contributed to albuminuria in HFD-KKAy mice.

We further examined diabetic mice lacking endothelial nitric oxide synthase (eNOS) by introducing the Akita mutation in the insulin2 (*Ins2*) gene^19^. This is a model of severe diabetes that recapitulates human DKD with mesangial expansion, mesangiolysis, glomerulosclerosis, and GBM thickening^19^. In this model, blood glucose level of eNOS-KO mice was 150 ± 15 mg/dl and this level was significantly increased at 460 ± 45 mg/dl at 6 months (*n* > 5). The urinary albumin level was also increased in eNOS-KO-DM mice (413 ± 107 μg/mgCr) compared to that in eNOS-KO mice (36 ± 4 μg/mgCr)^19^. The plasma PS level in eNOS-KO-DM mice was significantly increased (15.4 ± 2.73 μM, *n* = 5) compared with control eNOS-KO mice (5.97 ± 0.47 μM, Fig. 2i), although the renal function in both DM and non-DM mice was normal. Histological examination revealed that NOS-KO-DM diabetic mice showed significant changes in glomerulosclerosis compared with NOS-KO-control mice (Fig. 2j). These data further suggested that a higher PS level shows much severer glomerular lesion.

### PS is a predictor for incipient albuminuria in DKD patients

To confirm the relationship between PS and albuminuria observed in animal experiments, we recruited 362 diabetes patients from the U-CARE (urinary biomarker for continuous and rapid progression of diabetic nephropathy) study^20^. We analyzed two parameters: (i) the relationship between the baseline PS and albuminuria and (ii) the relationship between the baseline PS concentration and the progression of albuminuria up until 2 years later. We also measured soluble urokinase-type plasminogen activator receptor (suPAR), which associated with the incidence and progression of DKD by modification of podocyte barrier function^21,22^.

**Fig. 3:**
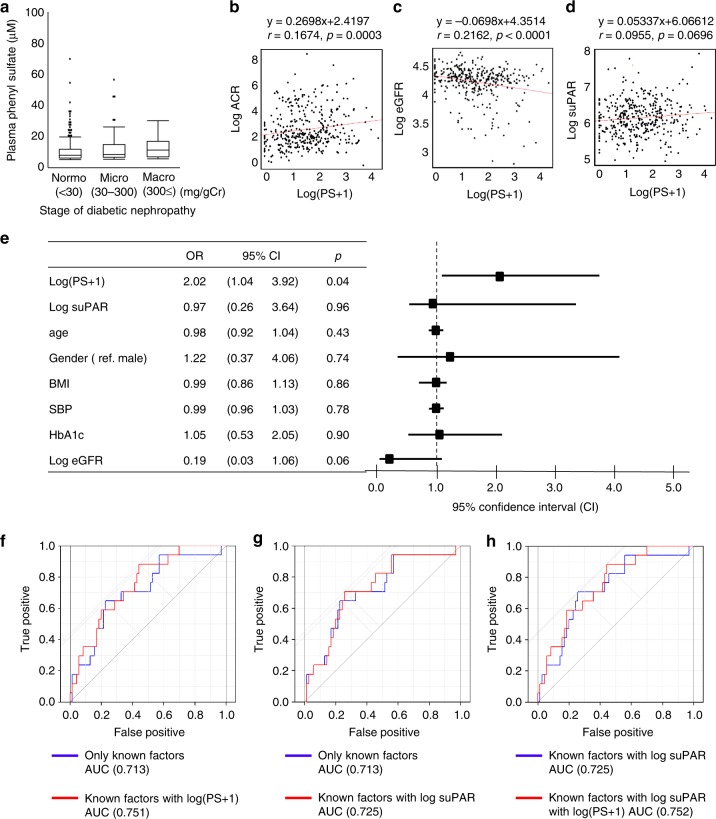
The clinical significance of PS in DKD patients. **a** The relationship between the plasma PS concentration (*n* = 362). **b** The relationship between the plasma PS concentration and the urinary albumin level (*n* = 362). **c** The relationship between the plasma PS concentration and the eGFR (*n* = 362). **d** The relationship between the plasma PS concentration and soluble urokinase-type plasminogen activator receptor (suPAR) (*n* = 362). **e** PS is a predictor of 2-year albuminuria. Adjusted odds ratio (95% confidence interval). Note that, among the known factors^23^, PS was the only factor that served as a predictor of the progression of 2-year ACR in patients with microalbuminuria (*n* = 87). Receiver operating characteristic (ROC) curve analysis in the U-CARE Study, showing comparison of known factors^23^. **f** AUC-ROC using known factors (0.713) and adjusted with PS (0.751) (*n* = 87). **g** AUC-ROC using known factors (0.713) and adjusted with suPAR (0.725). (*n* = 87). **h** AUC-ROC using known factors with suPAR (0.725) and adjusted with PS (0.752) (*n* = 87)

Participants in the U-CARE study (*n* = 362) had a mean age of 63.3 years, and 56.9% were male. The mean blood glucose concentration (BS) was 154.2 ± 56.4 mg/dl and the HbA1c was 7.2 ± 1.1%. The eGFR was 73.8 (17.1–115.4) ml/min/1.73 m^2^ and the albumin-to-creatinine ratio (ACR) was 11.0 (1.0–6407.4) mg/gCr. The serum PS level was 3.3 μM (0–68.1 μM) and suPAR was 460.8 (142–2740.2) pg/ml (Table 1). Supplementary Table 4 shows clinical data for patients with DKD based on the albuminuria and Fig. 3a shows the PS level.

In the Spearman rank-order test (Supplementary Table 5), the plasma PS level significantly correlated with ACR (Fig. 3b), eGFR (Fig. 3c), age, duration, HbA1c, and uric acid (UA), but not with suPAR (Fig. 3d). We next performed multiple regression analysis. To evaluate, we built three models: model 1 (crude model); model 2 (adjusted by known factors: age, gender, BMI, SBP, HbA1c, log (eGFR))^23^; and model 3 (full model; adjusted by model 2 plus other clinical factors in Supplementary Table 4, i.e., DBP, ALT, total cholesterol (TC), triglyceride (TG), high-density lipoprotein (HDL) and UA). Multiple regression analysis revealed that ACR was the only factor that significantly correlated with PS among all models (Table 2). Stepwise analysis for progression using known factors indicated the significance of the same factors. In addition, vice versa, analysis performed with ACR as an objective variable revealed that PS significantly correlated with the ACR among all models as well as stepwise analysis (Table 3). In this analysis, eGFR, gender, and blood pressure were also related to the ACR, as reported^24^, suggesting the validity of our cohort.

**Table 2:** Multiple regression analysis (objective value: (PS + 1))

	Model 1				Model 2				Model 3			
	Regression coefficient	95% CI		*p*	Regression coefficient	95% CI		*p*	Regression coefficient	95% CI		*p*
Log ACR^a^	0.107	0.043	0.171	0.001	0.078	0.009	0.146	0.028	0.076	0.007	0.145	0.031
Age^a^					0.009	−0.0001	0.018	0.053	0.009	−0.0004	0.019	0.061
Gender					−0.148	−0.333	0.038	0.119	−0.105	−0.303	0.092	0.297
BMI					−0.010	−0.032	0.013	0.395	−0.009	−0.034	0.016	0.488
SBP					−0.003	−0.009	0.003	0.337	−0.004	−0.011	0.004	0.345
HbA1c^a^					−0.049	−0.138	0.040	0.280	−0.066	−0.159	0.026	0.161
Log eGFR					−0.350	−0.758	0.058	0.093	−0.285	−0.720	0.149	0.199
Duration^a^									0.014	0.003	0.026	0.016
DBP									0.004	−0.008	0.015	0.520
ALT^a^									0.007	−0.001	0.015	0.105
TC									0.001	−0.002	0.005	0.503
TG									−0.001	−0.002	0.001	0.432
HDL									−0.001	−0.008	0.007	0.890
UA									0.028	−0.033	0.090	0.368

Multiple regression analysis based on clinical factors as an independent examined by variance inflation factor (VIF) <10. PS was used as an independent variable factor. Model 1 (crude model), model 2 (adjusted by known factors: age, gender, BMI, SBP, HbA1c, log (eGFR))^23^, and model 3 (full model, adjusted by model 2 plus fundamental clinical data in Supplementary Table 4 (DBP, ALT, TC, TG, HDL, and UA) were used.

*p < 0.05 was regarded as statistically significant. Remaining variables after the stepwise method based on Akaike’s information criterion (AIC) in model 3 are depicted as *

**Table 3:** Multiple regression analysis (objective value: Log ACR)

	Model 1				Model 2				Model 3			
	Regression coefficient	95% CI		*p*	Regression coefficient	95% CI		*p*	Regression coefficient	95% CI		*p*
Log(PS + 1)^*^	0.270	0.108	0.431	0.001	0.176	0.020	0.332	0.028	0.176	0.017	0.335	0.031
Age					−0.003	−0.017	0.011	0.652	−0.0004	−0.015	0.014	0.961
Gender^*^					0.333	0.056	0.611	0.019	0.396	0.097	0.695	0.010
BMI					0.020	−0.013	0.054	0.233	0.017	−0.022	0.055	0.397
SBP^*^					0.014	0.005	0.023	0.002	0.011	−0.0002	0.022	0.054
HbA1c					0.019	−0.115	0.152	0.786	0.035	−0.107	0.176	0.630
Log eGFR^*^					−1.919	−2.502	−1.335	<0.001	−1.883	−2.516	−1.250	<0.001
Duration									−0.003	−0.021	0.015	0.716
DBP^*^									0.010	−0.007	0.027	0.243
ALT									−0.004	−0.016	0.009	0.579
TC									−0.001	−0.006	0.005	0.761
TG									0.0004	−0.002	0.002	0.692
HDL									0.002	−0.010	0.013	0.795
UA									0.040	−0.054	0.134	0.403

Multiple regression analysis based on clinical factors as an independent examined by variance inflation factor (VIF) <10. ACR was used as an independent variable factor. Model 1 (crude model), model 2 (adjusted by known factors: age, gender, BMI, SBP, HbA1c, log (eGFR))^23^, and model 3 (full model, adjusted by model 2 plus fundamental clinical data in Supplementary Table 4 (DBP, ALT, TC, TG, HDL, and UA) were used

^*^p < 0.05 was regarded as statistically significant. Remaining variables after the stepwise method based on Akaike’s information criterion (AIC) in model 3 are depicted as *

Given the significant relationship between baseline PS and albuminuria, we examined logistic regression models to identify factors independently associated with the development of the 2-year ACR (Table 4). The mean follow-up duration was 2.1 ± 0.2 years (median = 2.1 years, interquartile range: 2.0–2.2 years). Based on model 1, PS and suPAR were the only factors related to the amount of change in the ACR during the 2 years, suggesting that PS and suPAR are independent risk factors for the ACR. In models 2 and 3, PS and suPAR were not significant. However, receiver operating characteristic (ROC) curve analysis revealed that the *c*-statistics value—the areas under the curve ROC (AUC-ROC) using known factors^23^ was 0.674 and the combination of PS increased the c-statistics value to 0.708 (Supplementary Fig. 8a). A combination of suPAR with known factors did not increase the *c*-statistics value from 0.674 to 0.664 (Supplementary Fig. 8b). However, the combination of PS with known factors, as well as suPAR, further increased the *c*-statistics value from 0.664 to 0.702 (Supplementary Fig. 8c).

**Table 4:** Logistic regression analysis

	Model 1				Model 2				Model 3			
	Odds	95% CI		*p*	Odds	95% CI		*p*	Odds	95% CI		*p*
Log(PS + 1)	1.42	1.02	1.98	0.040	1.27	0.88	1.84	0.202	1.34	0.91	1.95	0.135
Log SuPAR	2.42	1.27	4.59	0.007	1.42	0.66	3.08	0.371	1.39	0.61	3.14	0.435
Age					0.99	0.95	1.02	0.447	0.98	0.94	1.02	0.294
gender (ref. male)					1.18	0.61	2.28	0.624	1.32	0.65	2.68	0.447
BMI					1.01	0.93	1.09	0.866	1.00	0.91	1.09	0.976
SBP^*^					1.01	0.99	1.04	0.262	1.03	1.00	1.06	0.026
HbA1c					0.93	0.65	1.33	0.704	0.99	0.68	1.43	0.950
log eGFR^*^					0.10	0.03	0.32	<0.001	0.12	0.03	0.46	0.002
duration									0.99	0.95	1.04	0.729
DBP^*^									0.96	0.93	1.00	0.071
ALT									1.00	0.97	1.03	0.970
TC^*^									0.99	0.97	1.00	0.093
TG									1.00	1.00	1.01	0.279
HDL^*^									1.03	1.00	1.06	0.049
UA^*^									1.22	1.02	1.45	0.026

Two-year ACR deterioration shown by logistic regression analysis based on whole DKD. Model 1: only log(PS + 1) and Log suPAR (crude model), Model 2: Model 1 with known factors (age, gender, BMI, SBP, HbA1c and log eGFR)^23^, Model 3: Model 2 with other factors (duration, DBP, ALT, TC (total cholesterol), TG (triglyceride), HDL (high-density lipoprotein), and UA (uric acid)). The 95% confidence interval (95% CI) is listed

Remaining variables after the stepwise method based on the Akaike’s information criterion (AIC) in model 3 are depicted as *

Based on this, we performed stratified logistic analysis based on the disease stage. Owing to the insufficient number of macroalbuminuria patients for analysis, we examined the normoalbuminuria and microalbuminuria groups (Supplementary Table 6). In the normoalbuminuria group, PS was not related to the amount of change in the ACR during the 2 years in all models (Supplementary Table 6). On the other hand, in the microalbuminuria group, PS was the only factor related to the amount of change in the 2-year ACR in all three models, with an odds ratio of 2.02 (CI: 1.04–3.92) in model 2 (Fig. 3e and Supplementary Table 6). Stepwise model also indicated the significance of PS. However, suPAR was not related in either the normoalbuminuria or macroalbuminuria groups. ROC curve analysis also showed that the *c*-statistics value using known factors was 0.713 and the combination of PS increased this to 0.751 (Fig. 3f). A combination of suPAR with known factors also increased the *c*-statistics value from 0.713 to 0.725 (Fig. 3g). Further combination of PS with known factors and suPAR, still increased the c-statistics value from 0.725 to 0.752 (Fig. 3h). These data suggested that PS was related to ACR and that the PS concentration could predict the 2-year ACR deterioration in DKD patients, especially with microalbuminuria.

### Microbial enzymatic reduction of PS showed reno-protection

**Fig. 4:**
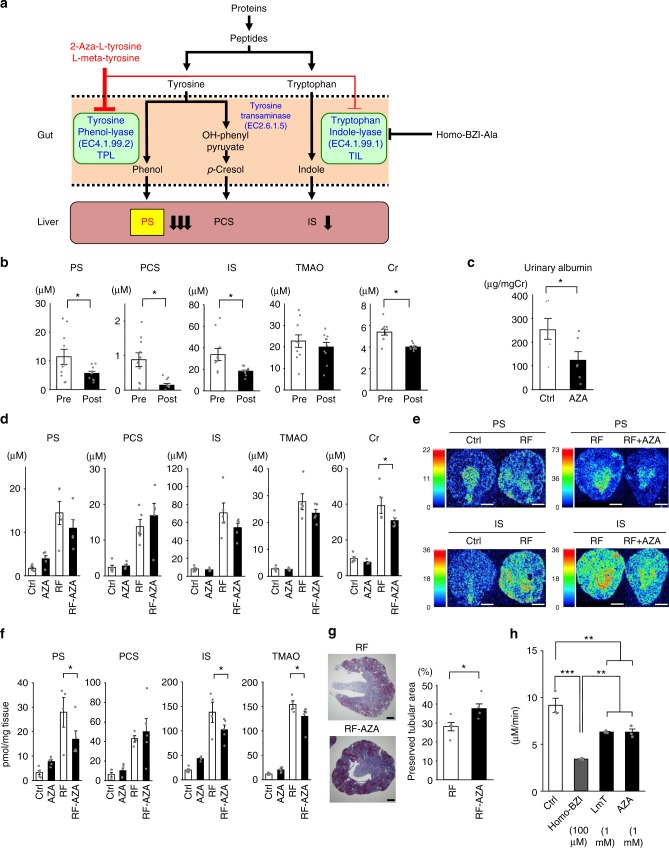
TPL inhibitors lowered PS concentrations and reduced renal damage in mouse models of diabetic kidney disease and adenine-induced renal failure. **a** Scheme of gut microbiome-derived PS, PCS, and IS generation. Dietary tyrosine is converted to phenol by tyrosine phenol-lyase (TPL) and further modified to produce PS or PCS. Dietary tryptophan is first converted to indole by tryptophan indole-lyase (TIL), further converted and finally modified into IS. Specific inhibitors of TPL are 2-aza-tyrosine and l-meta-tyrosine, while homo-BZI-Ala is a specific inhibitor of TIL. **b** Plasma concentrations of PS, IS, PCS, TMAO, and Cr in db/db mice before (Pre) and after treated with 2-aza-tyrosine (post, 10 mg/kg, *n* = 10). **c** Urinary albumin/creatinine ratio of db/db mice with either no intervention (Ctrl) or treatment with the TPL inhibitor 2-aza-tyrosine (AZA). *N* = 6 in each group. **d** Plasma concentrations of PS, IS, PCS, TMAO, and Cr in the adenine-induced renal failure mouse model treated with 2-aza-tyrosine (5 mg/kg) for 14 days. Four groups were measured: control mice (Ctrl), control mice treated with 2-aza-tyrosine (AZA), adenine-induced renal failure mice with no intervention (RF), and adenine-induced renal failure mice treated with 2-aza-tyrosine (RF-AZA). *n* = 5 in each group. **e** Representative imaging mass spectrometry spectra of PS and IS in kidney sections from renal failure mice (RF) and renal failure mice administered 2-aza-tyrosine (5 mg/kg) (RF + AZA). Mass spectrometry imaging shows the distribution of PS ([M-H]^−^, *m/z* 172) and IS ([M-H]^−^, *m/z* 212). Scale bar, 1000 μm. **f** Quantitative analysis of PS and IS tissue contents in mouse kidneys in RF (*n* = 4) and others (*n* = 5). **g** Morphometric analysis of the fractional cortical tubular area of Masson trichrome stained whole kidney images. *n* = 5 in each group. Data are presented as the percentage of the total cortex (*n* = 5). Scale bar, 500 μm. **h** Enzymatic activity of TIL. TIL activity under control conditions (Ctrl), with TIL inhibitor homo-BZI-Ala (100 μM) and a tyrosine phenol-lyase inhibitors (l-meta-tyrosine (LmT) or 2-aza-tyrosine (AZA), 1 mM), *n* = 3. *p < 0.05 vs. control according to Paired *t* test (**b**), data are mean ± SEM *p < 0.05 vs. control according to *t* test (**c**, **d**, **g**, **f**) or Tukey–Kramer test (**h**, *p < 0.05, **p < 0.01, ***p < 0.001). Source data are provided as a Source Data file

Nonlethal inhibition of microbial-specific enzymes has a therapeutic advantage, with lower selective pressure for the development of drug resistance^25^. Phenol is synthesized from dietary l-tyrosine by gut bacterial-specific tyrosine phenol-lyase (TPL, EC 4.1.99.2)^26^ and absorbed phenol is metabolized in to PS in the liver (Fig. 4a). TPL was reported to be expressed only in a minor population of *Enterobacteriaceae* (e.g., *Citrobacter*, *Proteus*, and *Erwinia*)^26^ and is not present in eukaryotes. Therefore, the idea arose that TPL inhibition might decrease the plasma PS level without any host toxicity. We synthesized the TPL inhibitors 2-aza-tyrosine^27^ and l-meta-tyrosine^28^ was purchased. We then examined their effects on the plasma PS level, albuminuria, and renal function in a diabetic model. Oral administration of 2-aza-tyrosine (10 mg/kg/day) for 1 week significantly decreased plasma PS, IS, and PCS in db/db mice (Fig. 4b). The increased albuminuria observed during the progression of diabetes was reduced by 2-aza-tyrosine (Fig. 4c, 10 mg/kg/day for 2 weeks). Furthermore, it was clearly demonstrated that the plasma creatinine (Cr) level was reduced by 2-aza-tyrosine (Fig. 4b), suggesting a renoprotective effect. Similarly, another TPL inhibitor, l-meta-tyrosine, also reduced the plasma PS and IS levels, although the Cr level was not significantly changed in db/db mice (Supplementary Fig. 9). These data suggested that 2-aza-tyrosine reduced not only albuminuria, but also the PS and IS levels, with a renoprotective effect in diabetic mice.

To confirm this renoprotective potency, we administered 2-aza-tyrosine to a mouse model of adenine-induced renal failure^29^. In this model, the plasma PS, IS, PCS, and TMAO levels were significantly increased (Fig. 4d). By administering 2-aza-tyrosine (5 mg/kg/day), the plasma Cr level was significantly decreased, with plasma PS and IS levels showing a strong tendency to decrease, but not statistically significant. Tissue PS and IS contents are significantly increased in the renal cortex of kidney failure mice shown by matrix-assisted laser desorption/ionization (MALDI) mass spectrometry imaging (MSI)^30^. Imaging also revealed that the tissue PS and IS levels were decreased by 2-aza-tyrosine treatment (Fig. 4e). The quantitative tissue contents analysis by LC/MS/MS clearly revealed that the PS, IS, PCS, and TMAO levels were increased by renal failure and that the accumulation of PS, IS, and TMAO were significantly reduced by 2-aza-tyrosine (Fig. 4f). Furthermore, the tubular area damaged by renal failure was improved by 2-aza-tyrosine (Fig. 4g). It is unclear how 2-aza-tyrosine reduced TMAO in renal failure tissue, but the amelioration of renal failure by 2-aza-tyrosine may have, in part, contributed to these beneficial phenomena.

The TPL-specific inhibitors 2-aza-tyrosine and l-meta-tyrosine reduced not only plasma and tissue PS levels, but also IS levels in the diabetic mouse model (Fig. 4b). PS is a metabolite of the phenol that is generated by TPL (EC 4.1.99.2) using tyrosine as a substrate. IS is generated by tryptophan indole lyase (TIL, EC 4.1.99.1) using tryptophan as a substrate^31,32^ (Fig. 4a). TPL and TIL enzymes show high-sequence homology^32^. It is conceivable that TPL inhibitors also partially inhibit TIL, resulting in a simultaneous reduction in PS and IS concentrations. To clarify this, we examined the effect of TPL inhibitors on TIL activity^33^. The specific TIL inhibitor 2-amino-4-(benzimidazol-1-yl)butyric acid (homo-BZI-Ala) at 100 μM, significantly inhibited TIL (apotryptophanase) activity as reported^33^ (Fig. 4h). Concentrations of 1 mM 2-aza-tyrosine and l-meta-tyrosine partly, though significantly, inhibited TIL activity. These data suggest that 2-aza-tyrosine inhibits both TPL and TIL, resulting in a dual reduction in plasma PS and IS concentrations followed by the recovery of renal damage.

### 2-Aza-tyrosine shows low-microbiota taxa pressure in RF mice

**Fig. 5:**
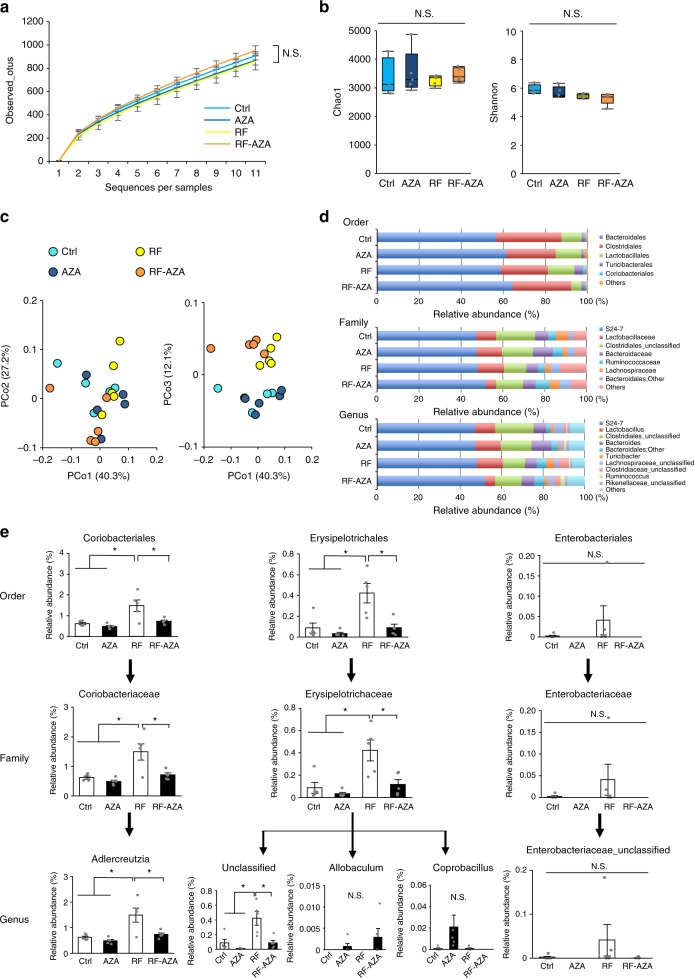
Effect of 2-aza-tyrosine (AZA) on the gut microbiome in adenine-induced renal failure mice. **a** Observed OTUs rarefaction analysis. OTU rarefaction curves of gut microbiome were used to estimate richness in the following groups: control mice (Ctrl); control mice treated with 5 mg/kg 2-aza-tyrosine (AZA); adenine-induced renal failure mice (RF); and adenine-induced renal failure mice treated with 5 mg/kg 2-aza-tyrosine (RF-AZA). **b** OTU-based α-diversity of each microbiome. No significant diversity was seen in any group by Chao1 (left) and Shannon (right) analyses. **c** Principal coordinates analysis of the microbiome profiles using weighted UniFrac. Scores are presented for the first principal component (PCo1) vs. both the second principal component (PCo2) and the third principal component (PCo3). **d** Relative abundance of microbiota based on the average number of each subgroup at order, family and genus levels. The major subgroups are indicated on the right. **e** The relative abundance of microbiota differed significantly among the four groups analyzed. The *y*-axis indicates the relative abundance of each microbe as a percentage. The proportional change in the minor groups correlated with renal failure and 2-aza-tyrosine treatment. The major microbial order (Bacteroidales, Clostridales, and Lactobacillales) were unchanged by either renal failure or 2-aza-tyrosine treatment. However, the minor populations (*Coriobacteriales* and *Erysipelotrichales*) were significantly changed in the correlation with the PS concentration by renal failure and 2-aza-tyrosine treatment. *n* = 5 in each group. Data were mean ± SEM. Statistical analysis was performed by the Tukey–Kramer test, followed by FDR correction of *p* values. *p_FDR_ < 0.1 was treated as statistically significant. The following groups were tested: control group (Ctrl); control group administered 2-aza-tyrosine (AZA); the renal failure group (RF); and the renal failure group administered 2-aza-tyrosine (RF-AZA). Metagenome Source data are provided as in a Source Data file

To further investigate the effect of 2-aza-tyrosine on the gut microbiota, we performed microbial 16S rRNA gene sequencing of feces. The α-rarefaction (observed OTUs) (Fig. 5a), α-diversity (Fig. 5b), and principal coordinates analyses using weighted UniFrac analysis (Fig. 5c) revealed that 2-aza-tyrosine did not cause a major shift in the major gut microbial composition. Next, we focused on the microbiome in which the number positively correlated with the plasma PS level in renal failure and was reduced by 2-aza-tyrosine. At the order level, the major population of gut microbiota (*Bacteroidales*, *Clostridales*, and *Lactobacillales*) became more pronounced at >90% and did not follow the criteria (Fig. 5d and Supplementary Fig. 10). However, two minor populations of microbial order (*Coriobacteria* and *Erysipelotrichales*) showed a significant increase in renal failure, and these increments were markedly decreased by 2-aza-tyrosine (Fig. 5e and Supplementary Fig. 10). At the family level, *Coriobacteriaceae* and *Erysipelotrichaceae* were significantly increased in renal failure and this rise was rescued by 2-aza-tyrosine (Fig. 5e and Supplementary Figs. 11 and 12). At the genus level, the composition of the 12 major genera was not altered in renal failure mice with or without 2-aza-tyrosine (Fig. 5e and Supplementary Figs. 13–15). However, *Adlercreutzia* and unclassified Erysipelotrichaceae positively correlated with plasma PS concentration, suggesting that these two minor taxa might, in part, influence the change in the plasma PS level. TPL activity was found in the Enterobacteriaceae family (*Citrobacter*, *Proteus*, and *Erwinia*)^26^, but in this experiment, the relative abundance of Enterobacteriaceae was very small (less than 0.1%) and no correlation was observed in the plasma PS concentration (Fig. 5e). We also found that, after 1 week of cessation of l-m-tyrosine (10 mg/kg) administration in db/db mice, the reduced plasma PS level recovered (Supplementary Fig. 16). These data suggest that TPL inhibitor did not significantly alter the major composition, but it reduced the PS production with a minor taxon modification.

**Fig. 6:**
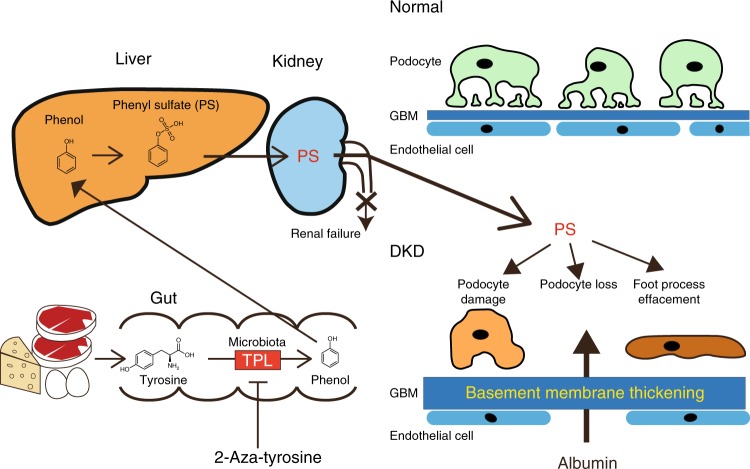
A schematic model of the generation and toxicity of PS. In gut microbiota, TPL converts tyrosine to phenol and ammonia. PS is also generated in the liver. PS accumulates in plasma as a metabolite and has deleterious effects on the vasculature and kidneys. In diabetic kidney disease, PS damages podocytes, accelerates GBM thickening, and induces proteinuria. Treatment with TPL inhibitors reduces plasma PS levels and prevents the progression of renal failure in animal models

Taken together, these data suggest that PS is a marker and a modifiable therapeutic target for DKD patients, especially those with microalbuminuria, and TPL inhibitor did not significantly alter the major composition, but it reduced the PS production with a minor taxon modification (summarized in Fig. 6).

## Discussion

It has been reported that the gut-derived metabolite PS is markedly increased with the progression of renal failure^34^ and is also higher in hemodialysis patients^35^. However, the precise biological activity or toxicity has not been clarified. The cause of DKD, includes irreversible (age, gender and duration of diabetes) and modifiable (hyperglycemia, hypertension and albuminuria) risk factors^36^. Intense multifactorial intervention targets glycemic control, blood pressure, including renin–angiotensin system blockade and dyslipidemia^37^. However, diabetic patients still have a poorer outcome^38^. The main pathophysiological changes in DKD kidney, include podocyte damage, thickening of the GBM, interstitial fibrosis, and hyalinosis of blood vessels^39^, as well as mitochondrial dysfunction in the podocytes^16^. We found that PS was toxic in podocytes and also reduced mitochondrial function. PS thickened the GBM and elicited inflammation with perivascular fibrosis. These multitoxicities of PS on the DKD kidney might lead to increased albuminuria. Therefore, DKD patients with a high-PS level should reduce the PS level to prevent the progression of albuminuria.

Although the limitations have been reported^40^, microalbuminuria is still a central target in the prevention and treatment of DKD^37^, and identifying which patients with microalbuminuria are most likely to develop more advanced kidney disease is important. We found that PS is related to the basal ACR and to the progression of albuminuria, suggesting the effectiveness of measuring PS in DKD patients with microalbuminuria. In addition, reducing circulating PS levels might also reduce the risk of DKD development (Fig. 6).

The circulating concentrations of uremic toxins is dependent on the variety of diet and time to draw blood^41^. To clarify the dietary effect of tyrosine on the PS concentration, we measured the PS levels in plasma collected serially over time in control (C57BL6) and diabetic (db/db) mice. After oral tyrosine administration, a greater increase in plasma PS level in diabetic mice occurred during the 4-h study period and peaked at 2 h compared with that of control (Supplementary Fig. 17). In addition, the serum PS level was higher in diabetic mice. The restriction of dietary protein is recommended for reducing glomerular hyperfiltration and pro-inflammatory gene expression in both CKD and DKD patients^42^. Our data suggests another aspect that reducing tyrosine intake in diabetic patients might decrease PS production, thereby reducing albuminuria and being renoprotective.

Concerning eGFR, our analysis uses only two time points of eGFR and the median eGFR decline was −1.8 in 2 years. Since the change in eGFR was small, we will need another cohort with a long observation period to reach a clear conclusion on the relation between PS and eGFR. In addition, under the absent decline in GFR, there is no role for circulating factor suPAR. The difference in the sensitivity between PS and suPAR may not be an issue with suPAR but rather with the cohort.

2-Aza-tyrosine reduced both PS and IS in the diabetic model. It is well-known that the charcoal adsorbent AST-120 significantly reduces PS and IS^35^. IS accelerates the progression of CKD^43^, but the contribution on albuminuria was limited^43^. Administration of IS to renal failure rats declined renal function, but the proteinuria was not changed^43^ In diabetic patients with normal renal function, AST-120 reduced the progression of renal dysfunction without changing IS level^44^. 2-Aza tyrosine decreased both PS and IS, the contribution of PS to albuminuria in db/db mice might be more potent than IS.

Our data showed that proteinuria was rapidly reduced in PS-loaded animals and we found that PS has a podocyte mitochondrial toxicity. It was reported that podocyte-specific loss of PHB2, a mitochondrial inner membrane structural protein led to early onset of proteinuria in 3-weeks and death after 5 weeks^45^. On the other hand, in our previous experiment, mitochondrial DNA knockout mice generate renal damage after 7–9 months^46^. The difference in onset may be attributed to differences in the model. We found that the combination PS and suPAR improved the *c*-statistics and this phenomenon raised another possibility of another mechanism of podocyte injury, e.g., suPAR is binding to and activating αv α3 integrins on podocytes, a process that leads to activation of small GTPase Rac-1 which in turns drives podocyte foot process motility and foot process effacement^47^.

The interference of gut microbiota may involve in the pathogenesis or development of diabetes^48^ and CKD^49^. However, little is known regarding DKD. It was reported a significant disruption of the epithelial tight junction in different CKD models^50^. In HFD-induced obesity and diabetes mice, expression of the tight junction proteins zonula occludens-1 (ZO-1) and occludin decreased^51^. We examined the epithelial tight junction in WT and transgenic rats and the expression levels of claudin I, occludin, and ZO-1 in the ileum and colon were unchanged (Supplementary Fig. 18).

We found that the microbial orders Coriobacteria and Erysipelotrichales increased in renal failure and decreased by 2-aza-tyrosine. Coriobacteriaceae are gram-positive anaerobic bacteria and in our previous study, the Coriobacteriaceae family and *Adlercreutzi*a were increased in renal failure mouse, but our intervention did not alter the increase^52^. It was reported that in CKD patients, the low-GFR group showed increased *Adlercreutzia*, which positively correlated with the TMAO level^53^. Erysipelotrichale are also gram-positive anaerobic bacteria and some types have been isolated from human feces and the bacteria encodes TPL^54^. Erysipelotrichales showed a decrease in Crohn disease patients^55^, but little has been clarified the relation to renal disease.

TPL has been found in a number of different strains of bacteria, most of which belong to the *Enterobacteriaceae* family, specifically to the genera *Citrobacter*, *Proteus*, and *Erwinia*^26^. However, the *Enterobacteriales* family and the Enterobacteriaceae family represent a minor population and little is known about the pathogenic contribution of such gut microbiota to human diseases. We also found that the microbial genus *Adlercreutzia* and Erysipelotrichaceae unclassified significantly correlated with a change in the PS levels. However, the TPL gene was not reported in the taxa according to the Genbank database search. Further experiments are needed to clarify the microbiome that produces phenol in renal failure.

The production of phenols and indoles by gut bacteria is associated with a variety of diseases in humans and animals. Phenols have been implicated as having a possible role in the causation of large bowel cancer^56^ and as causal factors in leukemia^57^. Elevated plasma levels of the phenol derivative 4-ethylphenylsulfate may promote anxiety-like behavior^58^. Chemical inhibition of TPL may also have a beneficial effect in preventing the phenol-induced disorders.

In conclusion, PS is not only an early diagnosis marker, but also a modifiable cause and therefore a target for the treatment of DKD. Chemical reduction of microbiota TPL should represent another aspect for developing drugs preventing DKD.

## Methods

### Materials

PS and ^13^C_6_ labeled PS were synthesized by Sundia MediTech (Shanghai, China) and ISOTEC (Sigma-Aldrich), respectively. 2-Aza-tyrosione^27^ and 2-amino-4-(benzimidazol-1-yl)butyric acid (Homo-BZI-Ala)^33^ were chemically synthesized. l-meta-tyrosine was purchased from Astatech (Bristol, PA). HUPEC^13^ were provided by Dr. Jeffrey B. Kopp (NIH, Bethesda). Human kidney proximal tubule cell line HK-2 (CRL2190) and Canine kidney cell line MDCKII (CRL2936) were purchased from ATCC.

### Animal experiments

The animal protocols were approved by the Tohoku University Institutional Animal Care and Use Committee (2016-007-1, 2-16-008-3). This study was conducted in accordance with the Guide for the Care and Use of Laboratory. SLCO4C1-Tg rats^6^ were bred at Tohoku University. C57BL6 mice, db/db mice and KKAy mice were obtained from CLEA Japan (Tokyo, Japan). Diabetes was induced in 8-week-old SLCO4C1-Tg rats^6^ by an intraperitoneal injection of STZ (50 mg/kg) dissolved in a 0.1 M citrate–phosphate buffer (pH 4.2)^59^. We estimated drug intake (PS, 50 mg/kg/day; 2-aza, 5 mg/kg/day; and l-meta-tyrosine, 10 mg/kg/day) by measuring the amount of water intake. For the renal failure model, 8-week-old C57BL/6N Jcl mice were fed a diet containing 0.2% wt/wt adenine for 6 weeks to induce tubular injury^29^, each group was further divided and 2-aza-tyrosine (10 mg/kg) was orally administrated for 2 weeks with normal chow. The urinary protein level was determined by enzyme-linked immunosorbent assay (ELISA) (Nephrat II, Exocell Inc., Philadelphia, PA). Plasma was collected from the experimental rats and the glucose, insulin, creatinine and BUN concentrations were measured according to the kit instructions (Wako Pure Chemical, Osaka, Japan).

### Histological examination

Kidney was fixed in 10% neutral buffered formalin and embedded in paraffin. Kidney sections were stained with periodic acid-Schiff (PAS), Masson’s trichrome, and Sirius red. For morphometric analysis of the glomeruli, PAS-stained sections were used for histomorphometric analysis. The whole-glomerular tuft volume was measured according to the reported method^60^. The area was quantified by measurement at ×200 magnification in 50 random nonoverlapping fields in each group (*n* = 4) using Image J (Figs. 1b and 2c). In SLCO4C1 TG rats, the preserved tubular area and fibrotic area (Sirius Red positive area) were quantified by measurement at ×20 magnification in whole renal cortex area in the field using Image J software (NIH, Bethesda, MD). In mice, the glomerular sclerosis area was measured using PAS-stained sections by Image J software, as reported^19^. In each analysis, 50 sections were examined from 3 animals in each examination. The preserved tubular area was also quantified by measurement at ×20 magnification in the whole renal cortex area in the field using Image J. The fibrotic area was quantified by measurement at ×200 magnification in 20 random nonoverlapping fields using Image J^61^. Electron microscopic analysis was performed as previously reported^17^. The mesangial matrix score was determined in at least 450 glomeruli from a total of 10 mice. Two pathologists, blind to the treatment of the individual rats or mice, performed the evaluation.

### Untargeted metabolomics

Plasma samples were taken from STZ-treated wild type rats: d7 (*n* = 6), d63 (*n* = 3), and d119 (*n* = 3). Plasma samples were also taken from STZ-treated transgenic rats: d63 (*n* = 3) and d119 (*n* = 4). Plasma samples were deproteinized by adding equal volumes of acetonitrile, then vortexed and centrifuged at 15,000×*g* for 5 min at room temperature and the supernatants were analyzed. UHPLC-QTOF/MS analysis was performed on an Acquit Ultra Performance LC I-class system (Waters Corp. Milford, MA) connected to a Waters Synapt G2-Si QTOF MS fitted with an electrospray ionization (ESI) source operated in the negative ion mode. Samples were separated using a Waters Acquity UPLC BEH Amide Column (1.7 μm, 2.1 × 150 mm) kept at 45 °C and a flow rate of 0.4 mL/min. Acetonitrile (10 mM ammonium bicarbonate (95:5, v/v) was used as mobile phase A and acetonitrile (10 mM ammonium bicarbonate (5:95, v/v) was used as mobile phase B. The gradient was applied as follows: 1% B at 0–0.1 min, 1–70% B at 0.1–6.0 min, 70–1.0% B at 6.0–6.5 min, and 1% B at 6.5–10.0 min. All data were processed using ProgenesisQI software (Nonlinear Dynamics, Newcastle, UK) for peak picking, alignment, and normalization to produce peak intensities for retention time (*t*_R_) and *m/z* data pairs. Features were identified from the Chemspider DB, Human Metabolome Database (HMDB) and Lipidmaps using the precursor and fragment ion spectra obtained by MS. The intensities of the identified features were imported to EZinfo software (Waters) for multivariate analysis, and their relative quantities were evaluated by PCA, PLS-DA, and OPLS-DA.

### Quantification of PS, PCS, IS, and TMAO by LC/MS/MS methods

PS, PCS, IS, and TMAO levels in animal plasma were measured as we recently reported^8^. Briefly, deuterated internal standards were used for all analytes and a TSQ Quantum Ultra triple quadrupole mass spectrometer (Thermo Fisher Scientific, Waltham, MA) equipped with a heated ESI source system was used. The mass spectrometer was operated in both positive and negative ionization modes. Samples were analyzed in single reaction monitoring mode by monitoring the ion transitions: *m/z* 173.0–>93.3 for PS. Data were acquired and analyzed using Xcalibur™ software (version 2.1, Thermo Scientific). For the human samples, the PS level in fresh-frozen plasma was measured by another method to help clinicians measuring PS by LSI Medience Co. (Tokyo, Japan). Briefly, LC–MS/MS data sets were acquired with a liquid chromatograph Nexera X2 LC0AD equipped with an ESI and triple quadrupole mass spectrometer 8050 (Shimadzu, Japan). The mass spectrometer was operated in the negative mode. The main parameters of the mass spectrometer are summarized in Supplementary Fig. 19. Data acquisition and analysis were performed using Shimadzu Lab Solutions version 5.89. The correlation of each measurement was confirmed with the correlation >0.99269 (Supplementary Fig. 19). The plasma levels of suPAR were measured by ELISA using the Quantikine Human suPAR Immunoassay kit (R&D Systems, Minneapolis, MN) according to the manufacturer’s instructions.

### Uptake experiment

Madin-Darby Canine Kidney (MDCK) II cells were stably transduced with SLCO4C1 or an empty vector and uptake experiments were performed as previously reported^9^. Uptake in HK-2 cells was initiated by adding 50 μM PS in the absence or presence of 100 μM ritonavir, a SLCO4C1 inhibitor. Uptake was terminated after a 10 min incubation. The concentration of PS in the cells was measured using LC/MS/MS. The uptake was calculated by dividing the uptake amount by the protein content of the cells.

### Cell toxicity assay

Differentiated HUPEC^13^ were cultured in RPMI1640 supplemented with 2% FBS and plated at 0.5 × 10^4^ cells/0.5 mL assay medium per well in 96-well collagen-coated culture plates and modified with the relevant culture condition, as reported^14^. After each compound was applied at the final concentration indicated, differentiated podocytes were cultured for 72 h and cell viability was measured by Cell Count Regent SF (Nacalai Tesque, Kyoto, Japan) as previously reported^17^. To measure the glutathione levels, a CellTiter-GloLuminescent Cell Viability Assay kit and a GSH/GSSG-Glo^™^ Assay kit were used (Promega, Madison, WI).

### Mitochondrial function measurement

Bioenergetic analysis of human cultured podocytes was carried out as previously described^17^. Briefly, the OCR and pH gradient (ECAR) of the fibroblasts from the Leigh patients or normal volunteers were measured using Seahorse XF 24 (Seahorse Bioscience). Cells were cultured in assay medium (70 mM sucrose, 220 mM mannitol, 10 mM KH_2_PO_4_, 5 mM mgCl_2_, 2 mM HEPES, 1.0 mM EGTA and 0.2% (w/v) fatty acid-free bovine serum albumin (BSA), pH 7.2) without CO_2_ for 60 min. After equilibration, cells were measured in the three respiration states followed by injections of three inhibitors of oxidative phosphorylation.

### Recruitment and measurement of the U-CARE cohort study

The Urinary biomarker for continuous and rapid progression of diabetic nephropathy (U-CARE) study^20^ is a multicenter, observatory clinical study that planned to investigate the urinary biomarkers in diabetic nephropathy (UMIN 00011525).

For diabetic nephropathy patient cohort study, we recruited 362 patients (U-CARE, registered UMIN 00011525). The analysis was approved by both the Tohoku University Ethics committee (2017-1-870) and Okayama University Ethics committee (1702-026). The collection of human samples was performed in accordance to the Tohoku University (2017-1-870) and after obtaining written informed consent adopted by the Okayama University (UMIN 00011525 and 1702-026). The study design and baseline characteristics were as follows: eligible participants were between 20 and 80 years of age with type 1 and type 2 diabetes and excluded people with liver cirrhosis and a new onset of malignancies within 5 years. Among 777 patients enrolled in 2012, 564 participants were eligible for enrollment in this study. An included criterion was that serum samples stocked in 2014 and clinical data between 2014 and 2016 would be available. Among the 564 participants, 362 patients with full data were selected. The phlebotomy protocol was as follows. Although blood at random visits could clot at room temperature for between 30 min and 2 h, samples were centrifuged, frozen at −80 °C and shipped on dry ice to the Okayama University Hospital, where the samples were aliquoted and frozen at −80 °C. Laboratory processing was performed at each enrolled hospital. Estimated GFR (eGFR) was calculated according to gender, age and the concentration of serum Cr according the Japanese equation Chronic Kidney Disease Epidemiology Collaboration (CKD-EPI)^62^. CKD-EPI is multiplied by a Japanese coefficient of 0.813; eGFR = 0.813 × 141 × min(SCr/κ, 1) α max(SCr/κ, 1)^−1.209^ × 0.993^Age^ × 1.018 [if female]α, where SCr is serum creatinine, *κ* is 0.7 for females and 0.9 for males, *α* is 0.329 for females and 0.411 for males, min indicates the minimum of SCr/ or 1, and max indicates the maximum of SCr/ or 1. Several baseline variables were measured as confounding factors. For BMI, some participants did not have information on BMI at baseline. To keep the sample size, we imputed an average of BMI measured 1 year before baseline and 1 year after baseline.

### Statistical analysis of clinical data in DKD patients

Parametric factors are presented as means and standard deviations or as medians and interquartile ranges. Nonparametric factors are presented as proportions by the result of Shapiro–Wilk test. As a result, skewed variables (eGFR, ACR, PS, and suPAR) were logarithmically transformed to improve normality prior to analysis. Since some PS values in the U-CARE study were calculated as 0 according to the measurement limit, we added 1 to calculate log transformed values. The correlation between the plasma PS level and various factors was calculated using the Spearman rank-order correlation.

To perform multiple regression analysis, we built 3 models as an independent variable examined by variance inflation factor (VIF) <10, and the plasma PS level and ACR were used as objective variables. Model 1 is the crude model. Model 2 is adjusted by known factors: age, gender, BMI, SBP, HbA1c, and log (eGFR))^23^. Model 3 is the full model, adjusted by model 2 plus other clinical factors in Supplementary Table 4 (DBP, ALT, TC, TG, HDL, and UA). Since blood sampling did not involve fasting and was strongly related to HbA1c, we did not adjust serum glucose concentration in the following analyses. A logistic regression analysis was also used to identify the factors independently associated with the development of 2-year ACR deterioration. We defined the increased ACR cases as follows^63,64^: normoalbuminuric at baseline: a case that progressed from normoalbuminuria to microalbuminuria or macroalbuminuria. Microalbuminuric at baseline: a case that progressed from microalbuminuria to macroalbuminuria or a case with ACR that doubled or more. Macroalbuminuric at baseline: a case with ACR that doubled or more. To identify the factors independently associated with the development of 2-year ACR, the same 3 multiple logistic regression models were used. Model 1: only log(PS + 1) and log(SuPAR) (crude model). Model 2: Model 1 with known factors (age, gender, BMI, SBP, HbA1c, and log(eGFR))^23^. Model 3: Model 2 with other factors (duration, DBP, ALT, TC, TG, HDL, and UA). Results are presented as odds ratios (ORs) with 95% CIs. ORs for all continuous variables were computed for each standard deviation (SD) change. ^*^p < 0.05 was set as statistically significant. The stepwise method was also performed based on the Akaike's information criterion (AIC).

### Microbiome analysis

The genomic DNA of gut microbiota was extracted from murine feces. The 16S rRNA genes in the DNA samples were analyzed using a MiSeq sequencer (Illumina, San Diego, CA)^29^. Microbial genomic DNA was extracted using a phenol–chloroform standard protocol, with vigorous shaking with 0.1 mm zirconia/silica beads. The V1–V2 region of the 16S rRNA genes was amplified from the extracted DNA using the bacterial universal primer set Rd1 SP sequence-27Fmod (5′-ACACTCTTTCCCTACACGACGCTCTTCCGATCT-AGRGTTTGATYMTGGCTCAG-3′) and Rd2 SP sequence-338R (5′-GTGACTGGAGTTCAGACGTGTGCTCTTCCGATCT-TGCTGCCTCCCGTAGGAGT-3′)^65^. Polymerase chain reaction (PCR) was performed with Tks Gflex DNA Polymerase (Takara Bio Inc.) and amplification proceeded with one denaturation step at 94 °C for 1 min, followed by 20 cycles of 98 °C for 10 s, 55 °C for 15 s and 68 °C for 30 s, with a final extension step at 68 °C for 3 min. The PCR products were purified using Agencourt AMPure XP (Beckman Coulter Inc.) and further amplified using a forward primer (5′-AATGATACGGCGACCACCGAGATCTACAC-NNNNNNNN-ACACTCTTTCCCTACACGACGC-3′) containing the P5 sequence, a unique 8 bp barcode sequence for each sample (indicated in N), and Rd1 SP sequence and a reverse primer (5′-CAAGCAGAAGACGGCATACGAGAT-NNNNNNNN-GTGACTGGAGTTCAGACGTGTG-3′) containing the P7 sequence, a unique 8-bp barcode sequence for each sample (depicted as N), and Rd2 SP sequence. After purification using Agencourt AMPure XP (Beckman-Courter), mixed samples were prepared by pooling approximately equal amounts of PCR amplicons from each sample^29^. Finally, MiSeq sequencing was performed according to the manufacturer's instructions. In this study, 2 × 300 bp paired-end sequencing was employed. As a result, 687,826 paired-end reads were obtained. To assemble the reads, fast length adjustment of short reads (v1.2.11)^66^ was used. 615,077 paired-end reads were assembled. Assembled reads with an average *Q* value <25 were filtered out using in-house script. The number of reads per sample was from 27,828 to 35,423. From each sample, 27,828 filter-passed reads were randomly selected and used for further analysis. Reads were then processed using quantitative insights into microbial ecology (QIIME) (v1.9.1) pipeline^67^. Sequences were clustered into operational taxonomic units (OTUs) using 97% sequence similarity and OTUs were assigned to taxonomy using the uclust method.

In microbiome analysis, *P*_FDR_ was calculated using the Benjamini and Hochberg false-discovery rate method (*α*  = 0.05) to adjust for independent multiple comparisons^68^. FDR calculation used the *p.adjust* function of the stats package of the *R* statistical language, version 3.4.2 (http://www.r-project.org/)^69^. *P_FDR_ < 0.1 was treated as statistically significant. JMP Pro software version 12 (SAS Institute Inc., Cary, NC) was used for statistical analysis.

### TIL (apotryptophanase) activity measurement

The assay was carried out in a cuvette (1 cm optical path length) in a BioSpectrometer Kinetic (Eppendorf, Hauppauge, NY) at 37 °C as previously reported^70^, but with some modification. The reaction mixture contained 0.005% BSA (Sigma), 1 mM reduced glutathione (Sigma), 100 μM pyridoxal phosphate (Sigma), 100 μM NADH (Sigma), 3 U/mL lactate dehydrogenase (Sigma) and apotryptophanase (10 U/mL, Sigma) in 0.15 M potassium phosphate buffer (pH 8.0). Following a 3 min incubation with inhibitor, the enzymatic reaction was initiated by adding l-tryptophan (300 μM final, Sigma). The decrease in optical density at 340 nm was monitored for 5 min at 5 s intervals and used for calculating the TIL reaction velocity. The inhibitory effect of TPL inhibitors (2-aza-tyrosine and l-meta-tyrosine) on TIL enzymatic activity was measured. Homo-BZI-Ala was used as a positive control of TIL inhibitor.

### Mass spectrometry imaging of kidney sections

MSI of kidney sections was performed as previously reported^30^. Briefly, kidney tissues were sectioned to 8 μm in thickness with a cryostat and thaw-mounted onto indium tin oxide-coated glass slides. Using a 0.3 mm caliber nozzle airbrush (Procon boy FWA Platinum; Mr. Hobby), 9-AA (750 μL, 4.5 mg/mL in methanol) was sprayed on the slide. MALDI-MSI analysis was performed using iMScope (Shimadzu, Japan). Mass spectra of the designated areas on a specimen photographed before matrix application were acquired in the negative ion mode. Mass spectra were acquired under the condition of a laser frequency and scanning mass ranging from *m/z* 200 to *m/z* 220 and *m/z* 170 to *m/z* 189. The spatial interval of data points was 50 μm, giving 10,120 data points in total for each section. The data collected through the microscopic system were digitally processed using Imaging MS Solution analysis software (Shimadzu).

### Quantitative PCR analysis

Whole kidneys were homogenized in TRIzol reagent (Invitrogen) and extracted according to the manufacturer’s directions. The cDNA synthesis was performed using a transcri ptor first strand cDNA synthesis kit (Roche). Primers purchased from Applied Biosystems are shown in Supplementary Table 10. The sequences of the primers and probes are certificated by the company, but not open based on the company policy.

### Western blot

Western blotting of ileum and colon were performed as previously described^61^. The primary antibodies against claudin 1 (1:1000 dilution, ab15098), occludin (1:1000 dilution, ab31721), and ZO-1 (1:1000 dilution, abG041) were purchased from Abcam.

### Statistical analysis

The data are expressed as means ± SEM or SD. *p < 0.05, **p < 0.01, and ***p < 0.001 were considered statistically significant. JMP Pro software version 12 (SAS Institute., Cary, NC) was used for the statistical analyses.

### Reporting summary

Further information on research design is available in the Nature Research Reporting Summary linked to this article.

## Supplementary information

Supplementary Information

Description of Additional Supplementary Files

Supplementary Data 1

Reporting Summary

Source Data

## Data Availability

The data that support the findings of this study are available from the corresponding author upon reasonable request. The source data underlying Figs. 1a, b, 1g, 1j, 2a, b, 2d–g, 2i, j, 4b–d, 4f–h, 5a–e, and Supplementary Figs. 1, 3–7, 9, 16–19, Supplementary Tables 2 and 3 are provided as a Source Data file. Untargeted metabolome analysis underlying Fig. 1c–f, Supplementary Fig. 2a–d and Supplementary Table 1 are provided in a file named Supplementary Data 1. The microbiome data were deposited in the DDBJ database (https://trace.ddbj.nig.ac.jp/DRASearch/) under accession number DRA006253. A Reporting Summary for this Article is available as a Supplementary Information file.
