# Supplementary material for: Gut microbiome-derived phenyl sulfate contributes to albuminuria in diabetic kidney disease
Source: Nat Commun. 2019 Apr 23;10:1835. doi: 10.1038/s41467-019-09735-4 (PMC6478834; doi:10.1038/s41467-019-09735-4)
Supplement: Supplementary file 1 — Supplementary Information [file 41467_2019_9735_MOESM1_ESM.pdf]

Supplementary Information

Gut microbiome-derived phenyl sulfate contributes to albuminuria in diabetic kidney disease

Kikuchi K. et al.

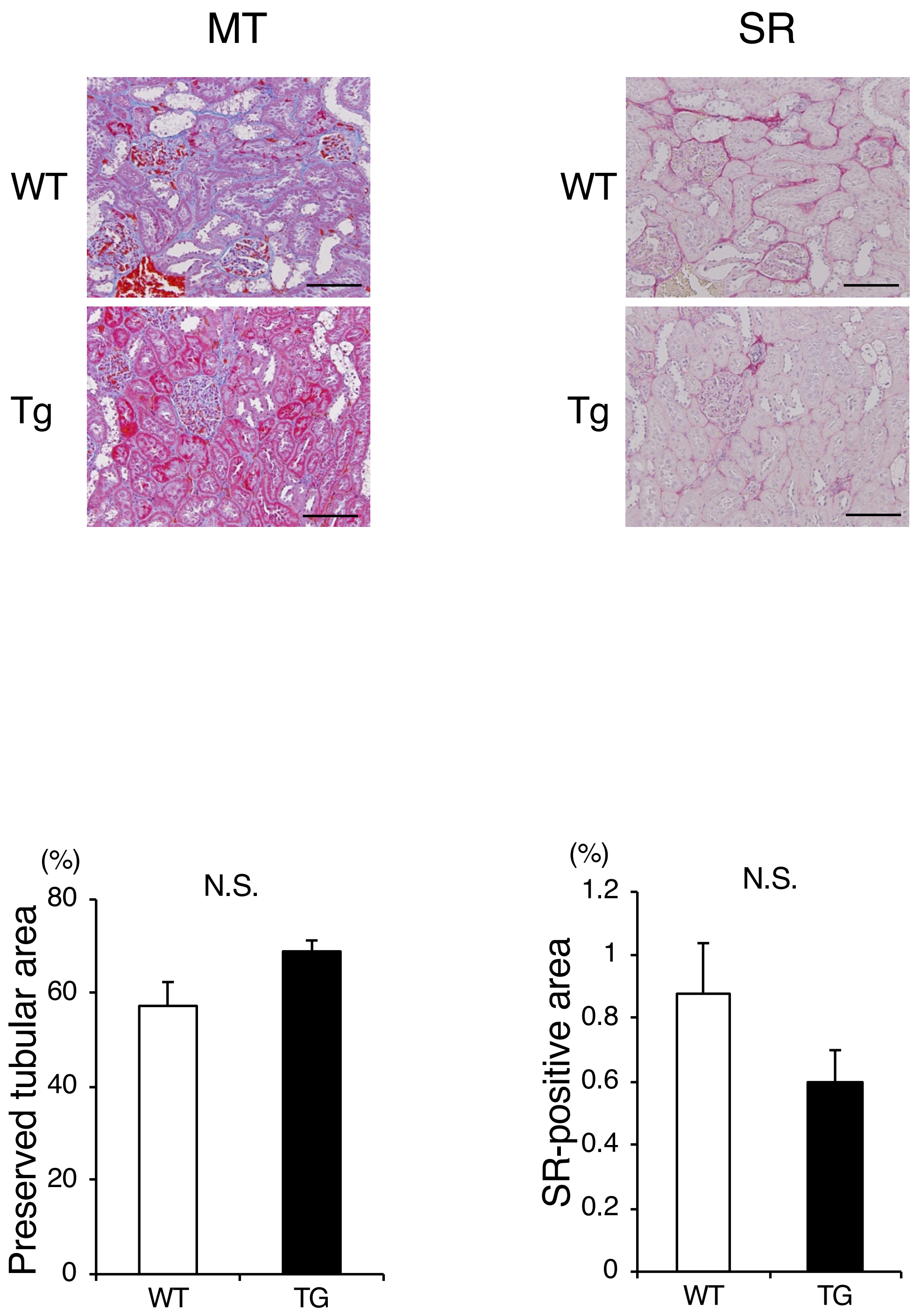

Supplementary Fig. 1

Histological analysis of wild type (WT) and transgenic SLCO4C1 (TG) rats under diabetic conditions. Masson trichrome (MT) and Sirius red (SR) staining showed no significant difference in tubular area and fibrotic area between WT and TG rats. Scale bars were 200 μm.

WT (n=5) and TG (n=6). Data are expressed as mean ± SEM. Statistical analysis was performed Student-*t* test.

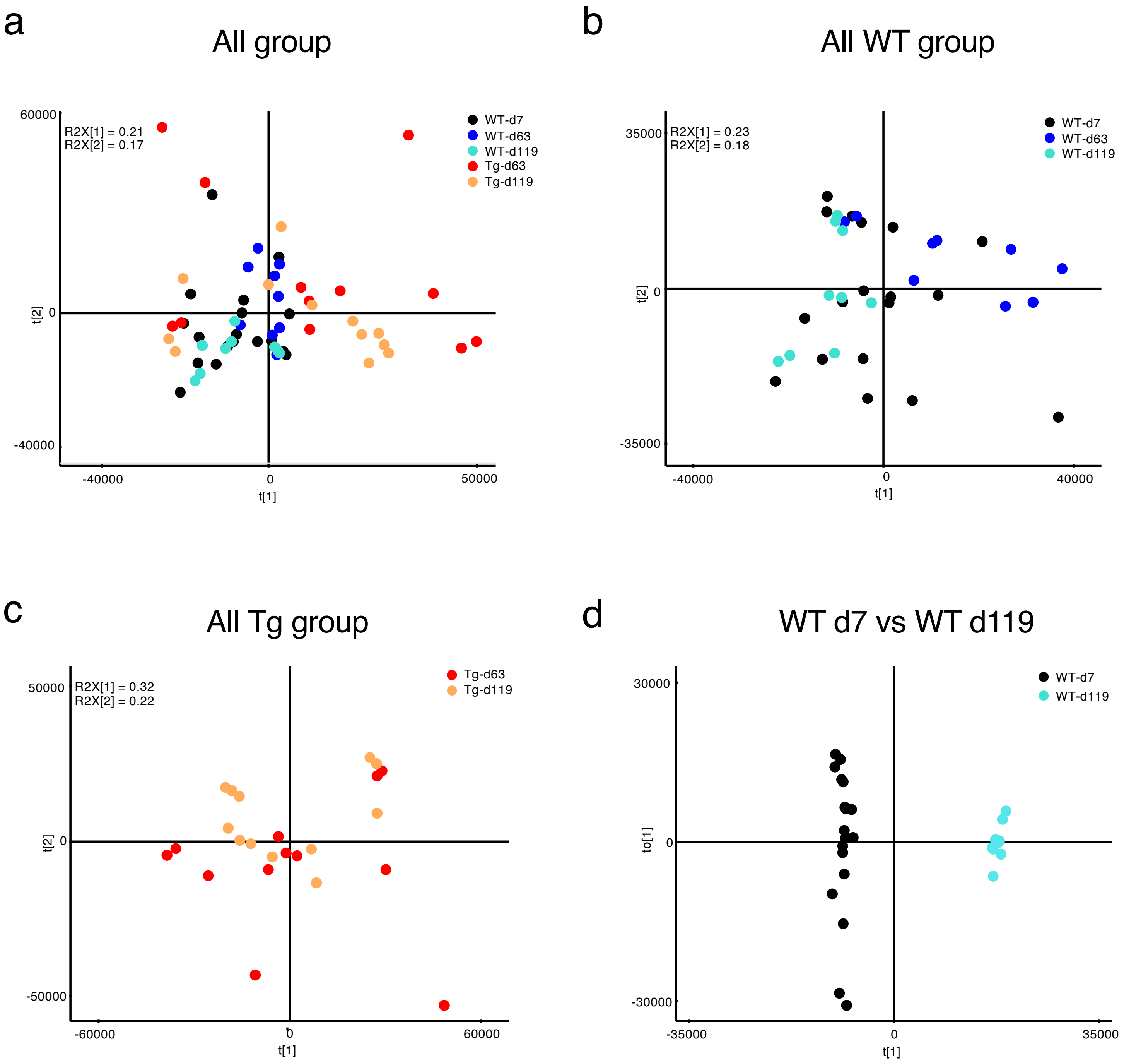

Supplementary Figure 2

Principal component analysis (PCA) scores plot and orthogonal partial least square-discriminant analysis (OPLS-DA) of wild type (WT) and transgenic (Tg) rats.
(**a**) Variation among the five groups in the PCA scores plot based on the chemical features detected in the plasma samples by metabolic profiling. Each group is color coded as follows: WT-d7 = black circles, WT-d63 = blue circles, WT-d119 = cyan circles, Tg-d63 = red circles and Tg-d119 = orange circles. (**b)** Variation among the WT groups in the PCA scores plot based on the chemical features detected in the plasma samples by metabolic profiling. (**c**) Variation between the Tg groups in the PCA scores plot based on the chemical features detected in the plasma samples by metabolic profiling. (**d**) Variation between WT-d7 and WT-119 in the OPLS-DA analysis based on the chemical features detected in the plasma samples by metabolic profiling.

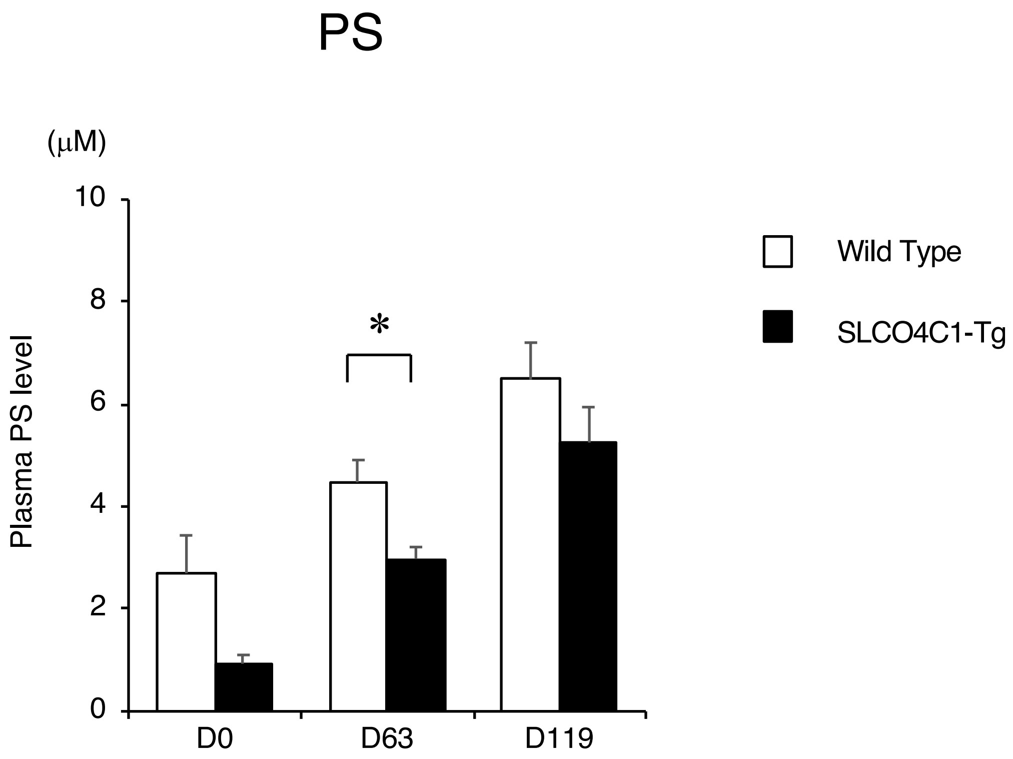

Supplementary Figure 3
PS levels in plasma samples from wild type and transgenic SLCO4C1 rats were measured by LC/MS/MS. The data are expressed as mean ± SEM. Statistical analysis was performed by the Student's *t*-test. *$p<0.05$ was treated as statistically significant.

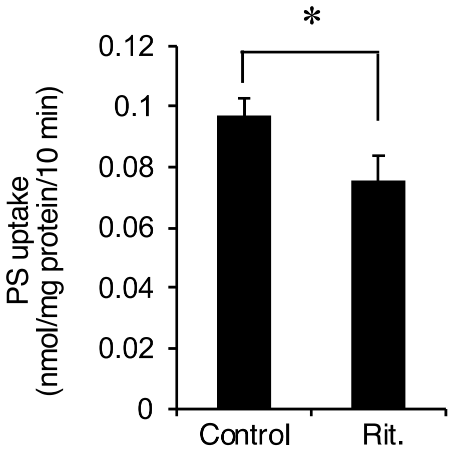

Supplementary Figure 4

Uptake study in HK-2 cells. PS uptake by HK-2 cells was significantly inhibited by 100 mM of the SLCO4C1 inhibitor ritonavir (Rit). Control (n=3) and Rit. (n=3). Mean ± SEM, *p<0.05, Student's *t*-test.

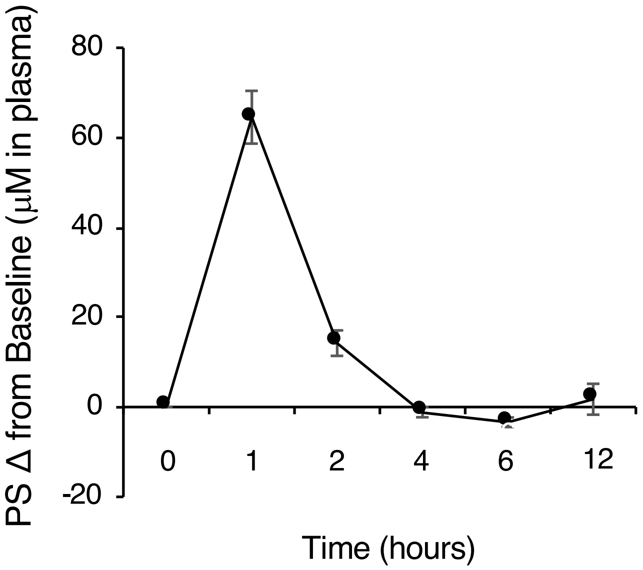

Supplementary Figure 5

Circulatory PS levels after PS administration. PS (50 mg/kg) was given orally by gavage to db/db mice (20 weeks old) after overnight fasting and blood samples were collected and measured. n=3 in each group. The data are expressed as mean ± SEM.

a

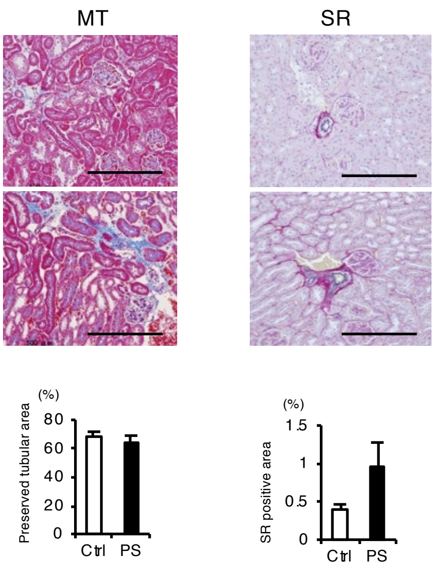

b

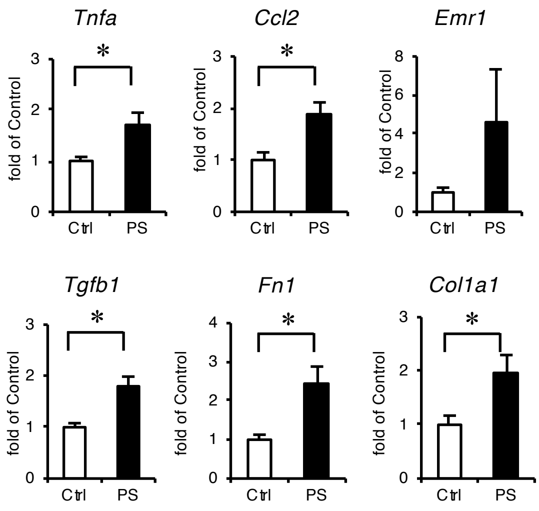

Supplementary Figure 6
(**a**) Histological analysis in db/db mice that were either untreated or treated with PS. Masson trichrome (MT) and Sirius red (SR) staining revealed some fibrotic area, whereas the reduced tubular area was unaffected in the PS treated group. Scale bars = 500 μm.
(**b**) Quantitative PCR. The mRNA levels of *Tnfa*, *Ccl2*, *Emr1*, fibrogenic *Tgfb1*, *Fn1* and *Col1a1* in db/db mice either untreated (Ctrl, n = 5) or PS-treated (PS, n=6) were measured by real-time PCR. The mRNA expression levels were quantified by densitometry and normalized with GAPDH. The data are expressed as mean ± SEM. Statistical analysis was performed by the Student's *t*-test. *p<0.05 was treated as statistically significant.

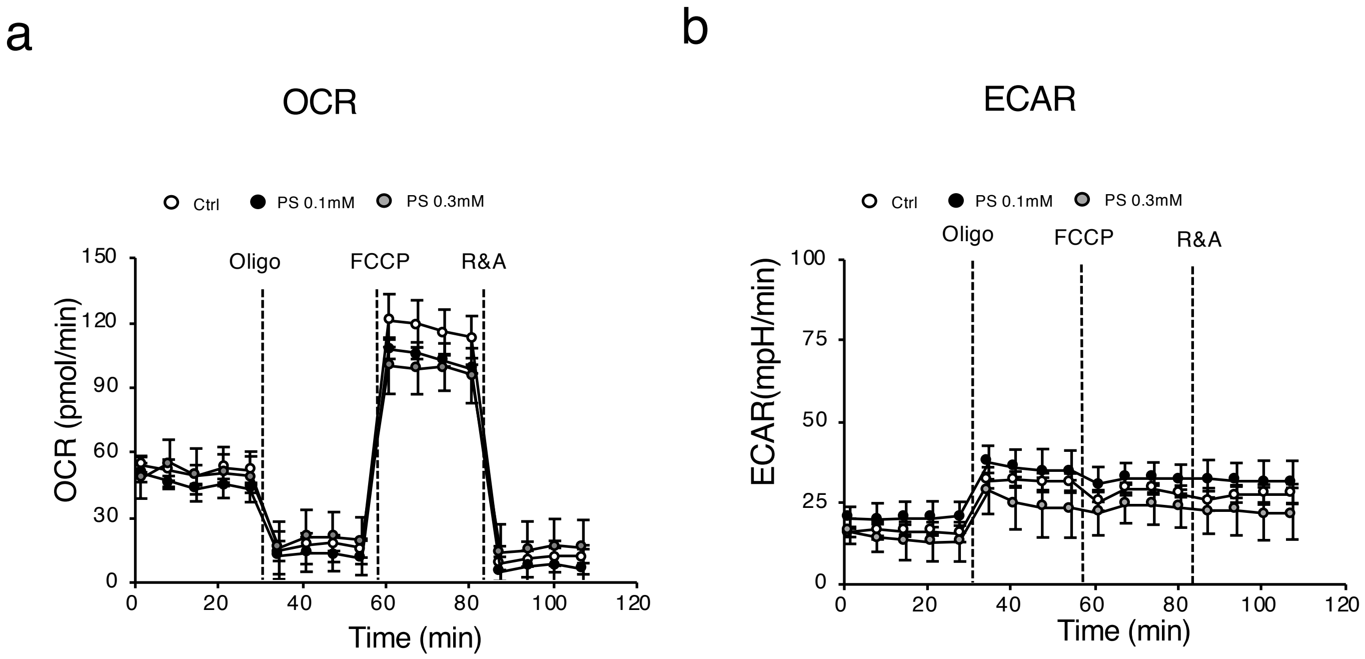

Supplementary Figure 7

Bioenergetic examination of oxygen consumption rate (OCR) (**a**) and extracellular acidification rate (ECAR) (**b**) in HUPEC cells measured by extracellular flux analyzer. Cells were untreated (Ctrl, white circles), treated with 0.1 mM PS (black circles) or treated with 0.3 mM PS (grey circles); n=3 in each case. The data are expressed as mean ± SEM. Oligomycin (1 μM), carbonyl cyanide-p-trifluoromethoxyphenylhydrazone (10 μM), and rotenone with antimycin (1 μM) are indicated as oligo, FCCP and R &A, respectively.

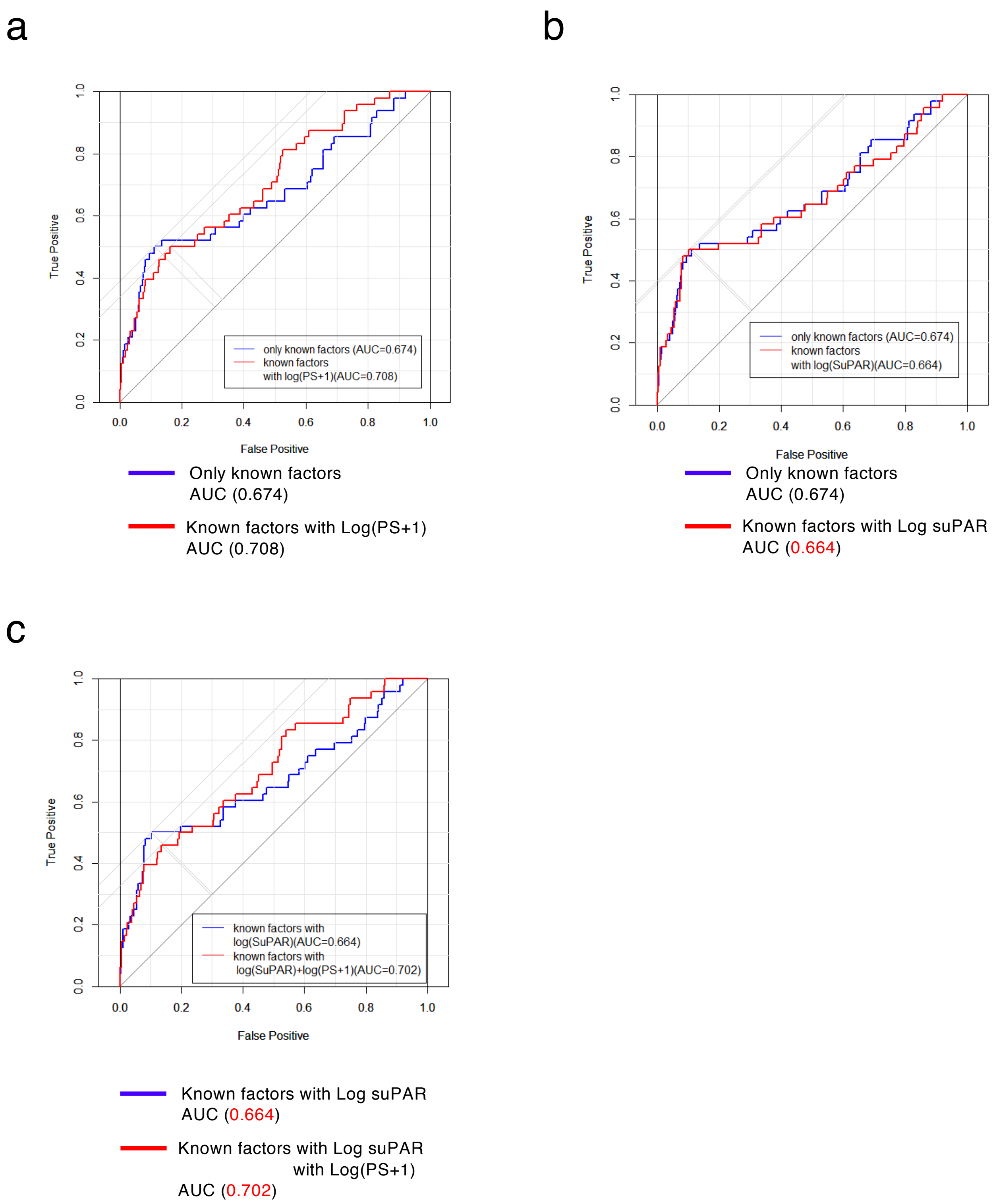

Supplementary Figure 8

Receiver operating characteristic (ROC) curve analysis of the whole U-CARE Study.
ROC curve analysis in 2-year albuminuria compared with known factors.
(**a**) AUC-ROC using known factors (0.674) and adjusted with PS (0.708). (**b**) AUC-ROC using known factors (0.674) and adjusted with suPAR (0.664). (**c**) AUC-ROC using known factors with suPAR (0.664) and adjusted with PS (0.702).
Known factors: Age, Gender, BMI, SBP, HbA1c, Log eGFR

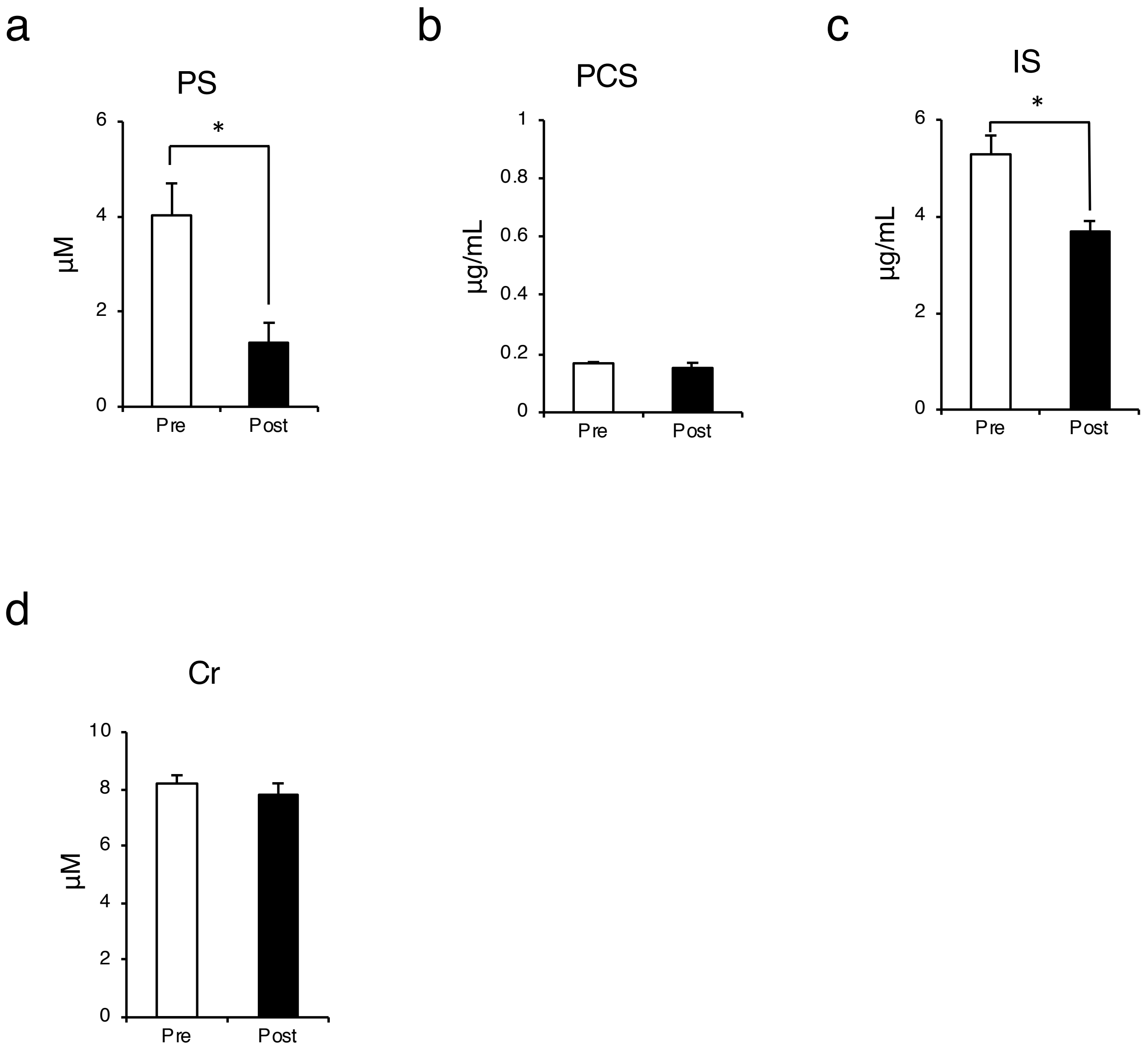

Supplementary Figure 9

Plasma level of uremic toxins and creatinine in db/db mice orally administered L-m-tyrosine (10 mg/kg/day) for 14 days. The plasma levels of PS and IS were significantly reduced by L-m-tyrosine treatment. Pre-administration (Pre, white column) and post-administration (Post, black column) results are shown; n=10 in each case. The data are expressed as mean ± SEM. Statistical analysis was evaluated by the paired t-test. *$p<0.05$ was treated as statistically significant.

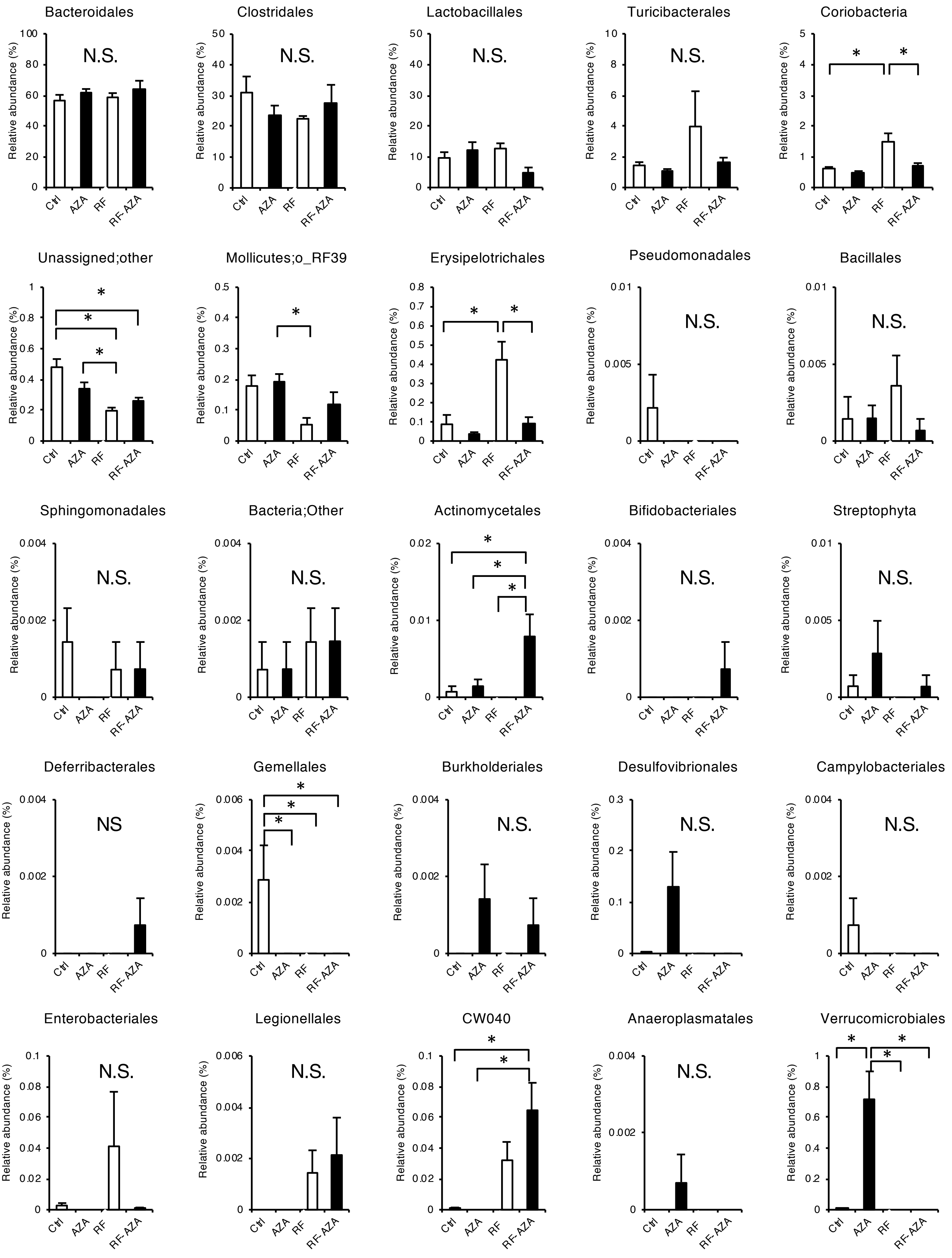

Supplementary Figure 10

Microbial ribosome 16S rRNA gene sequencing and analysis of the feces from normal and renal failure mice with or without 2-aza-tyrosine treatment. Data show the taxa at the order level. Statistical analysis was performed by the Tukey-Kramer test. *p<0.05 was treated as statistically significant.

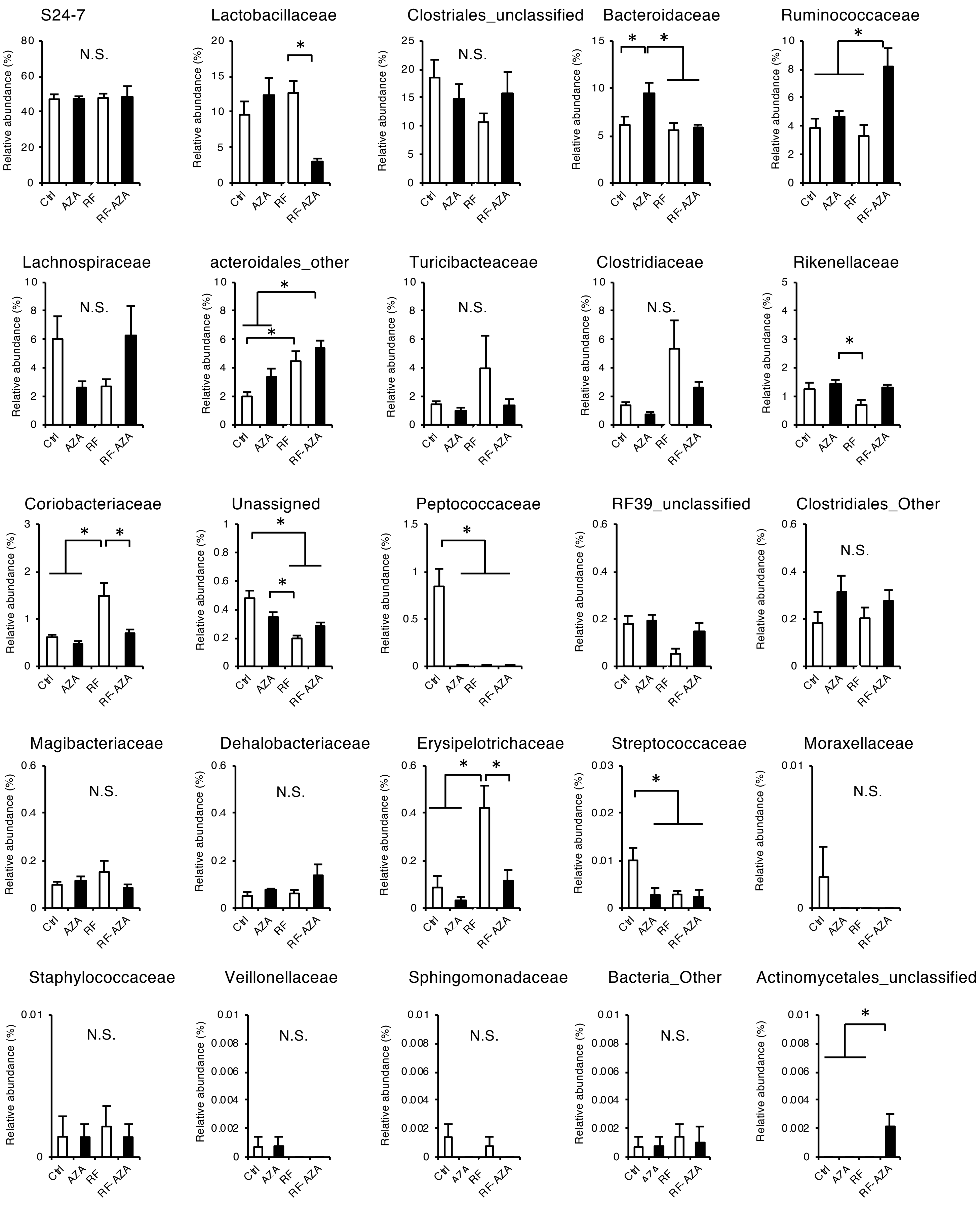

Supplementary Figure 11

Microbial ribosome 16S rRNA gene sequencing and analysis of the taxa at the family level. Statistical analysis was performed by the Tukey-Kramer test. *$p<0.05$ was treated as statistically significant.

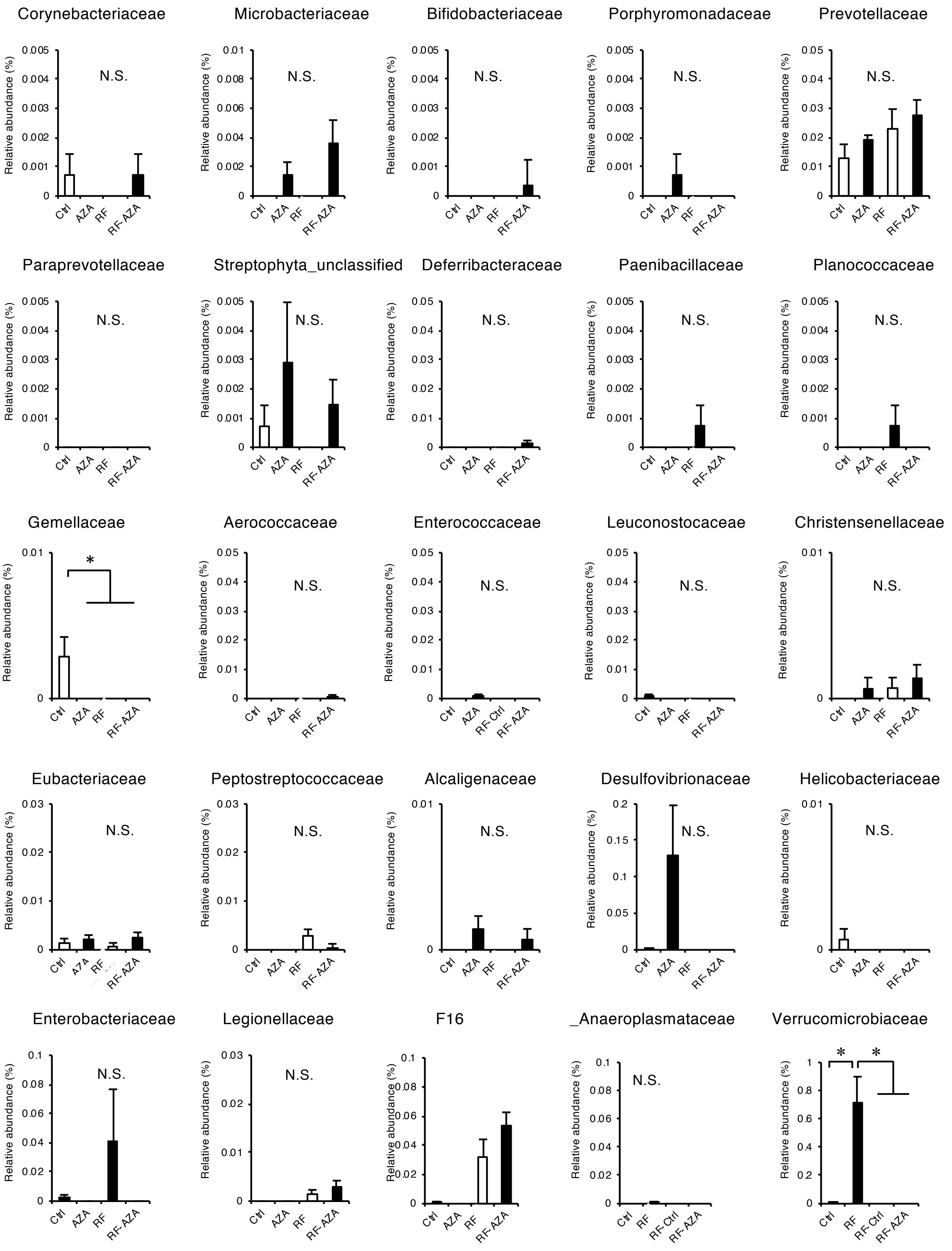

Supplementary Figure 12

Microbial ribosome 16S rRNA gene sequencing and analysis of the taxa at the family level. Statistical analysis was performed by the Tukey-Kramer test. *p<0.05 was treated as statistically significant.

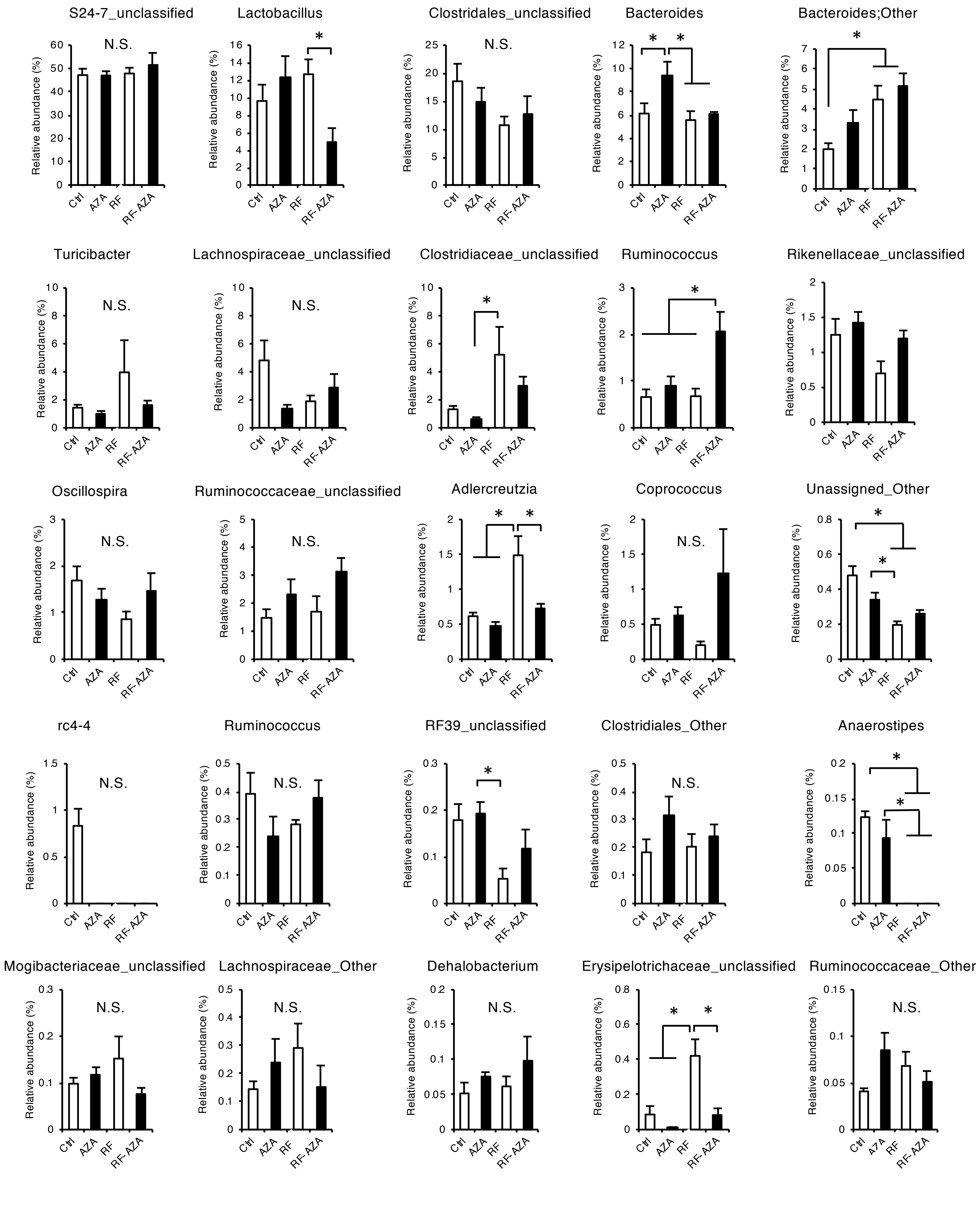

Supplementary Figure 13

Microbial ribosome 16S rRNA gene sequencing and analysis of the taxa at the genus level. Statistical analysis was performed by the Tukey-Kramer test. *p<0.05 was treated as statistically significant.

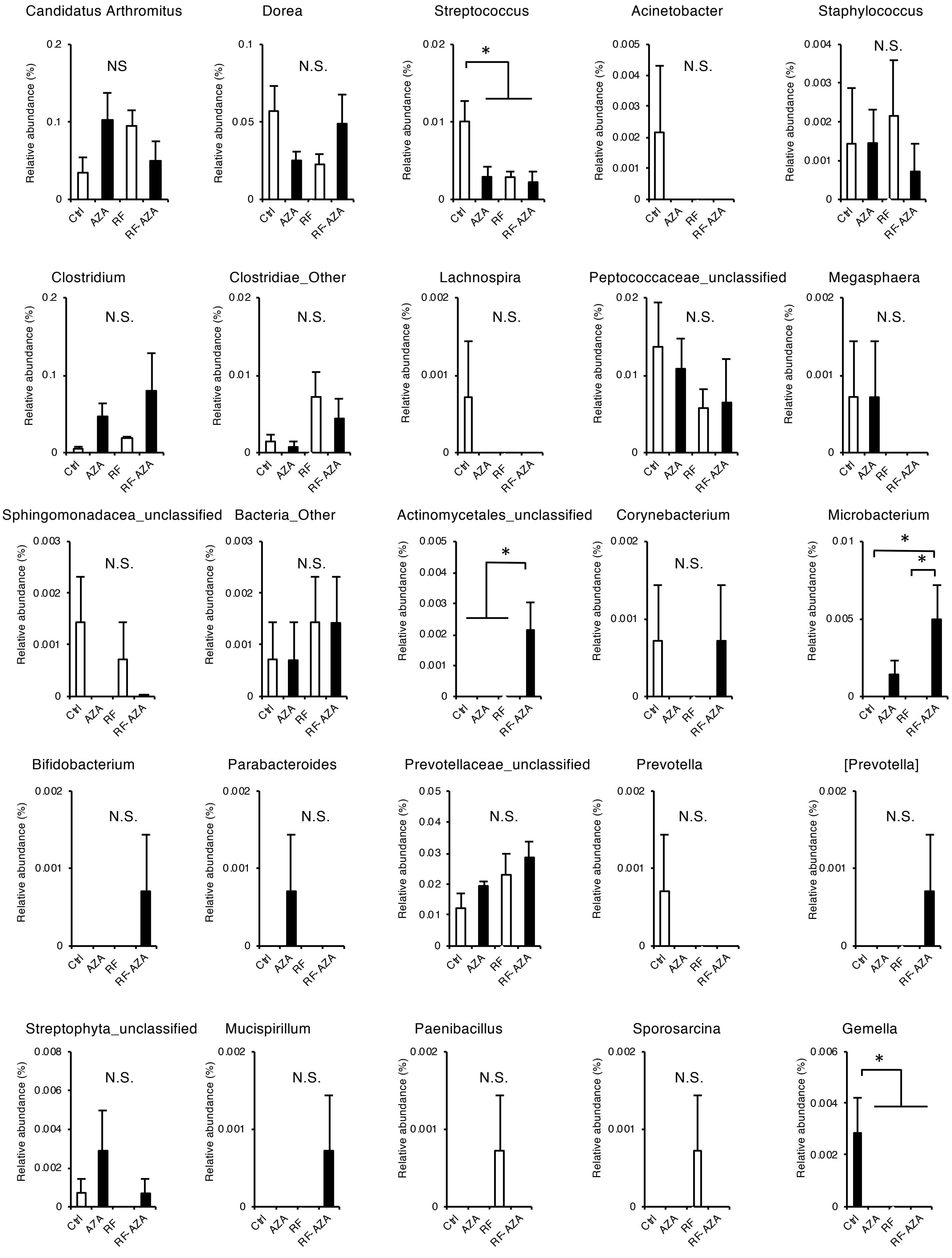

Supplementary Figure 14

Microbial ribosome 16S rRNA gene sequencing and analysis of the taxa at the genus level. Statistical analysis was performed by the Tukey-Kramer test. *p<0.05 was treated as statistically significant.

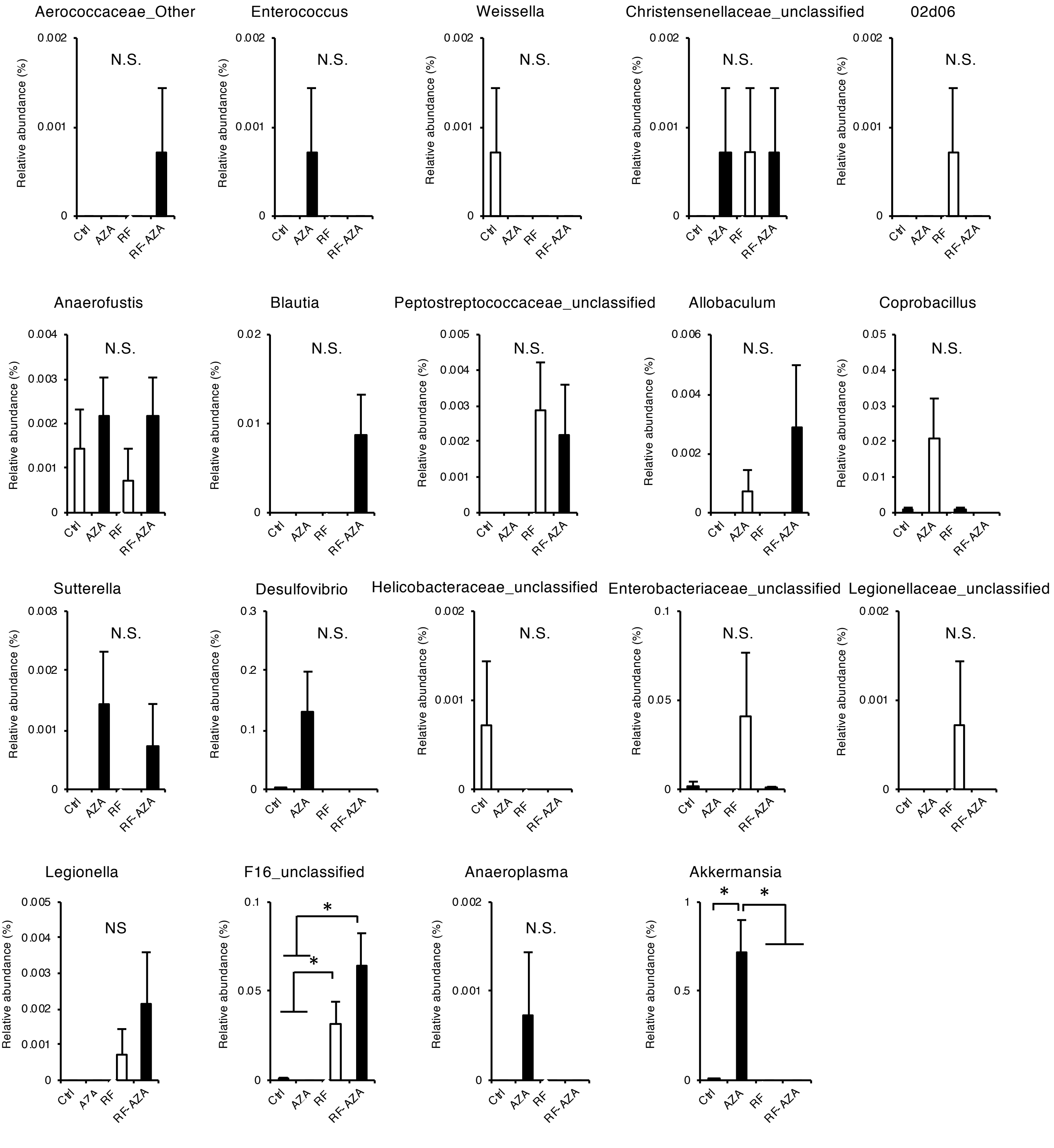

Supplementary Figure 15

Microbial ribosome 16S rRNA gene sequencing and analysis of the taxa at the genus level. Statistical analysis was performed by the Tukey-Kramer test. *p<0.05 was treated as statistically significant.

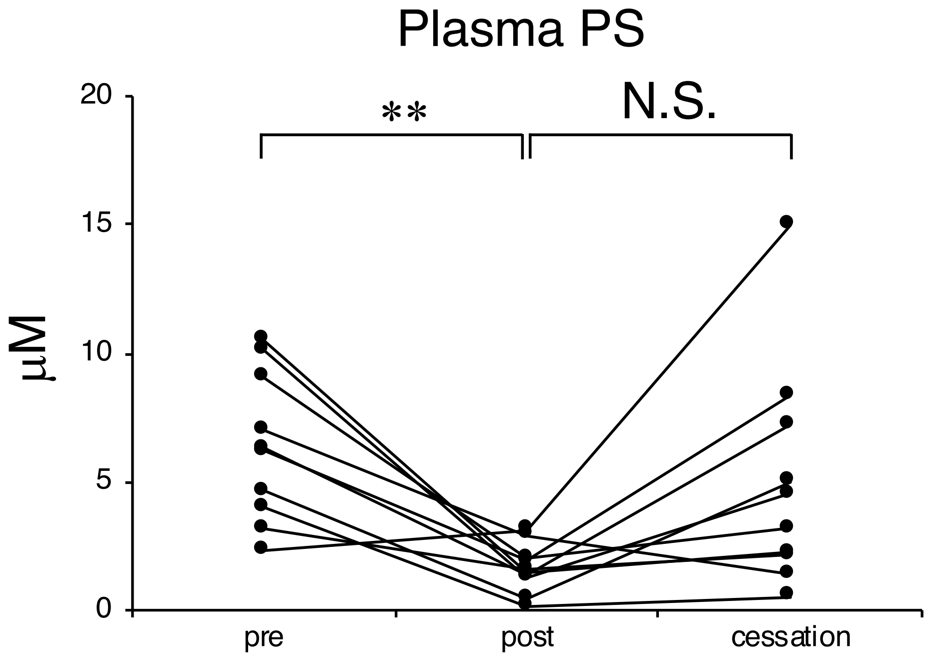

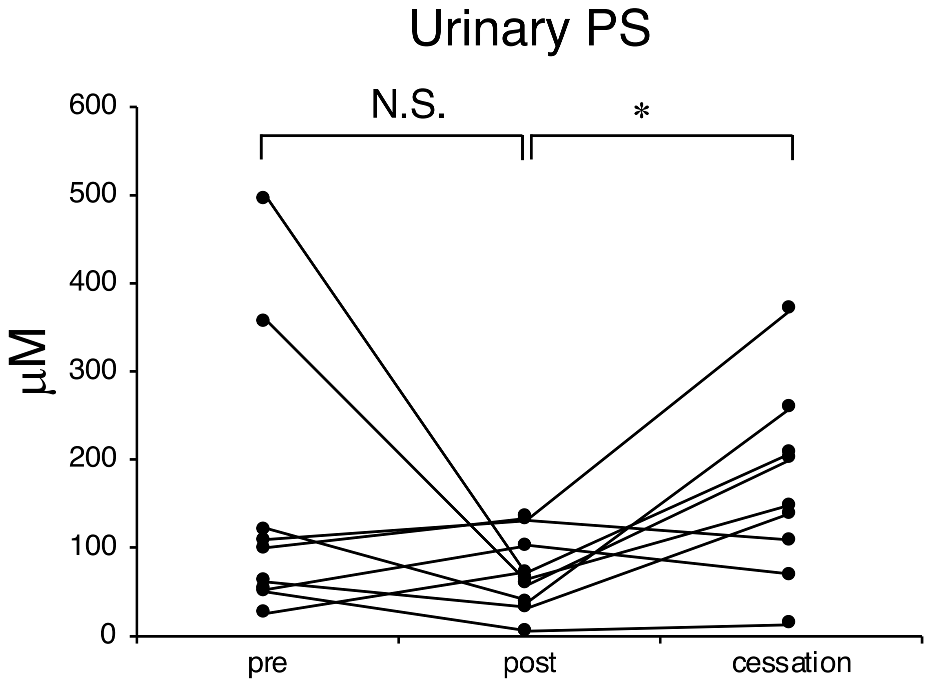

Supplementary Figure 16

Plasma and urinary levels of PS in db/db mice. PS levels were significantly reduced by L-meta-tyrosine treatment (10 mg/kg/day) and this reduction was recovered by the cessation of L-meta-tyrosine treatment for one week. Statistical analysis was evaluated by the paired t-test with Bonferroni correction. *p< 0.05/2=0.025 and **p<0.01/2=0.005 were considered to be statistically significant compared to post-administration.

The PS level was significantly reduced by L-m-tyrosine administration (10 mg/kg/day) and the reduction was recovered by cessation of L-m-tyrosine treatment for one week.

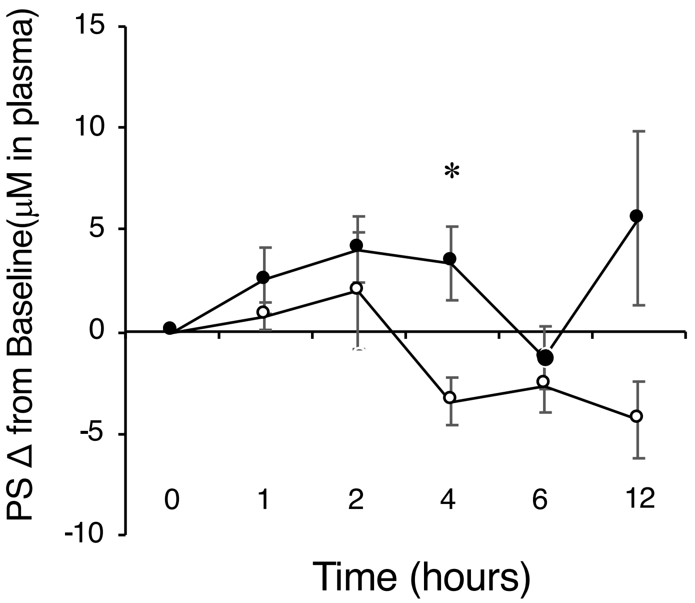

Supplementary Figure 17

PS levels after tyrosine administration. Effects of tyrosine on PS concentrations in plasma collected serially over time from C57BL6 (20 weeks old) and db/db (20 weeks old) mice; n = 7 in each group). Data are expressed as changes from the study baseline (0 min). The data are expressed as mean ± SEM. Statistical analysis was performed by the Student's *t*-test. *p<0.05 was treated as statistically significant.

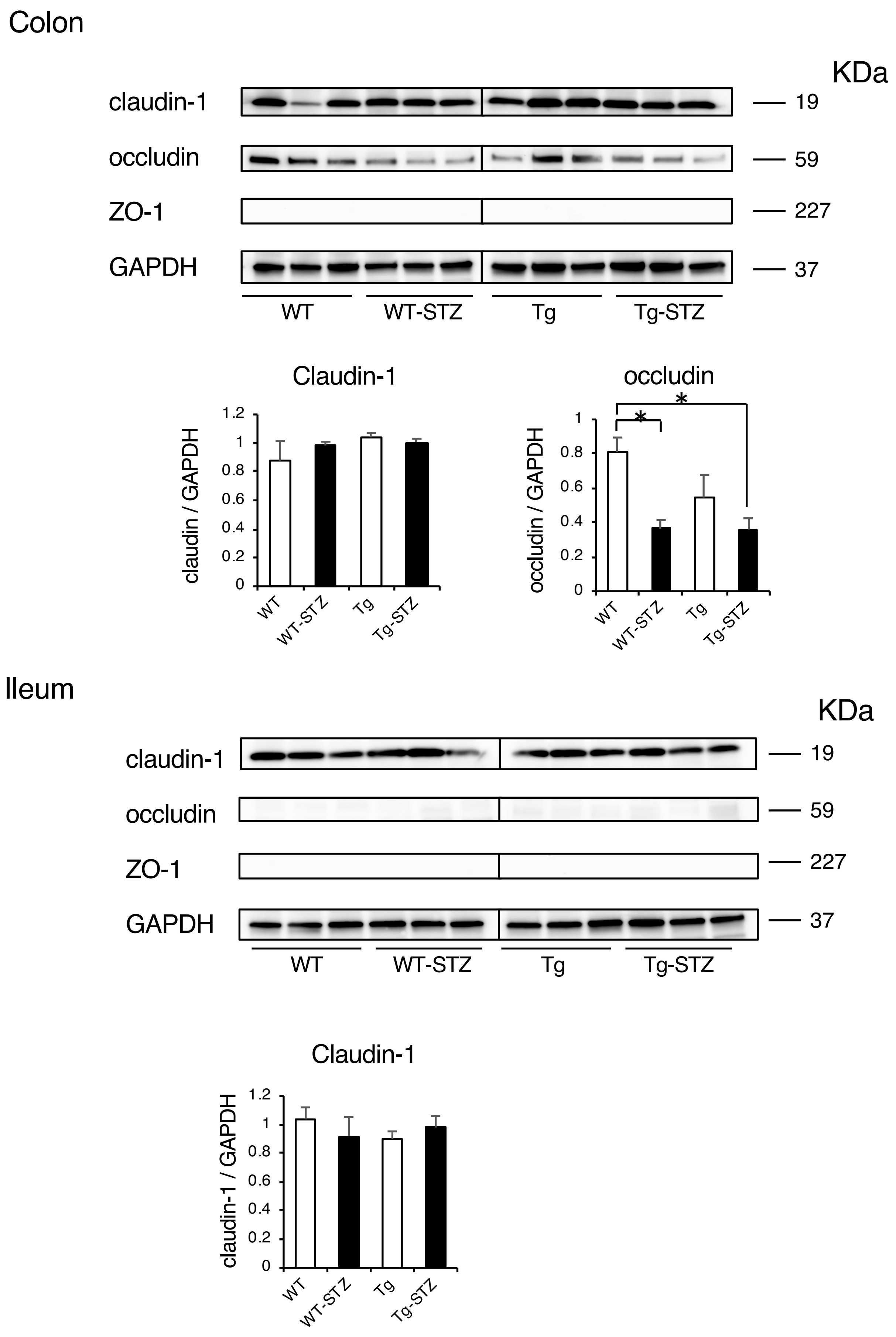

Supplementary Figure 18

Western blot analysis of ileum and colon in SLCO4C1-Tg rats. The proteins from ileum and colon were obtained from streptozotocin-treated SLCO4C1 Tg rats after 8 weeks of streptozotocin (STZ) injection.

Data was shown by mean ± SEM. *p<0.05, Tukey-Kramer test.

a

MRM (Multiple Reaction Monitoring) parameters for the determination of PS and internal standard.

Analyte	Mass transitional (m/z)	Collision Energy (V)	Q1/Q3 Resolution
Phenyl sulfate (PS)	173.0> 80.00	18.0	Unit
4-Acethylphenyl sulfate	215.0> 135.05	22.0	Unit

b

Correlation of PS values analyzed by large and small scale methods.

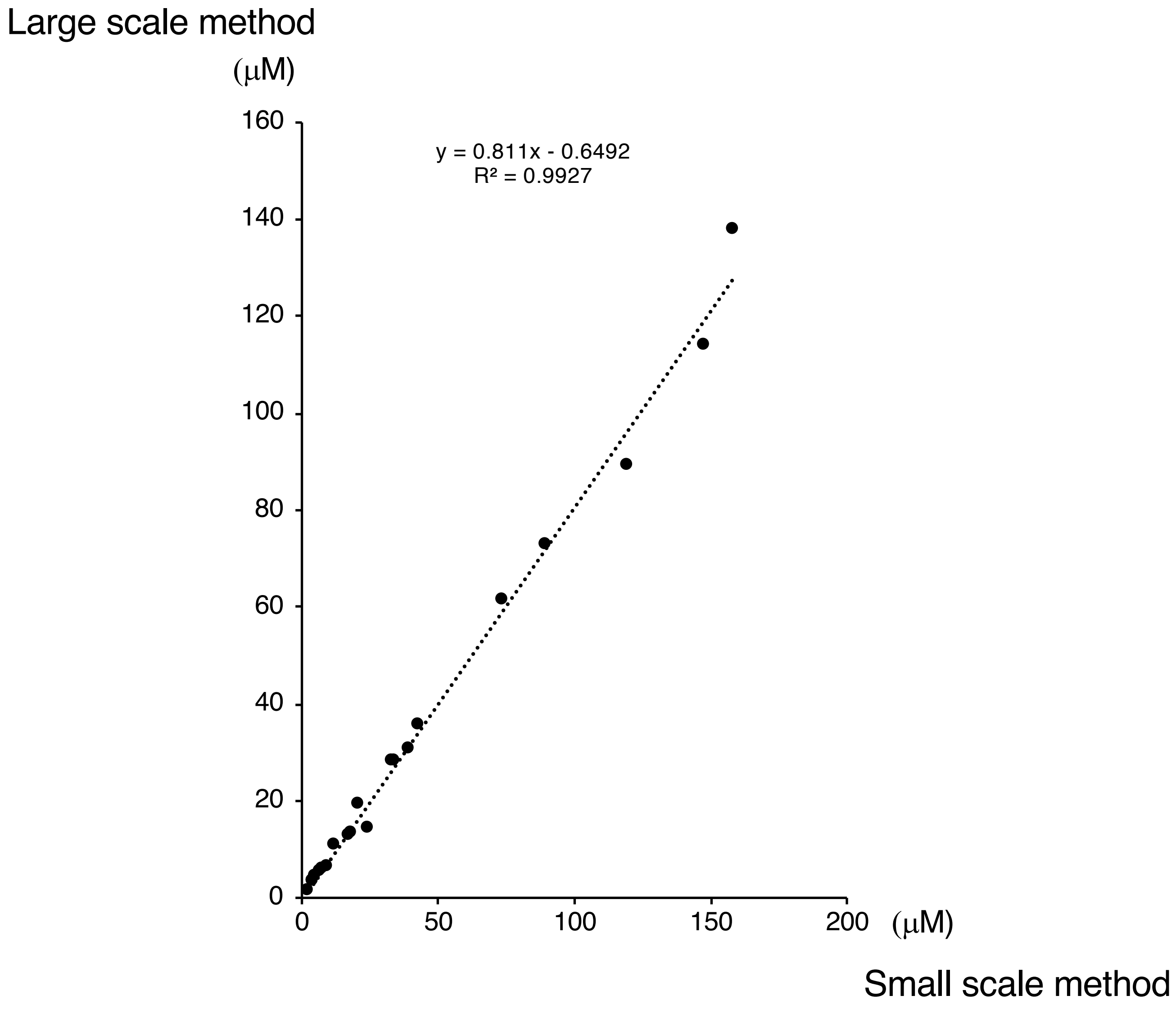

Supplementary Figure 19
Correlation of PS values analyzed by the large scale-based and the small scale-based methods. **(a)** Multiple Reaction Monitoring (MRM) parameters for the determination of PS and internal standard. **(b)** Correlation of PS values analyzed by small scale in mouse and large scale in human.

m/z	RT (min)	WT d7 v.s WT d119				WT d63 v.s Tg d63		WT d119 v.s Tg d119	
		VIP Score	Anova (*p*)	Fold Change	Minimum CV%	Fold Change	Anova (*p*)	Fold Change	Anova (*p*)
172.97	0.31	5.90	3.9E-10	1.9	12.9	1.7	6.3E-06	1.5	1.4E-06
461.32	0.68	1.40	1.1E-08	1.7	10.9	1.5	7.5E-04	1.4	1.3E-02
445.32	0.48	1.34	9.8E-08	2.1	8.8	2.4	5.3E-02	1.4	3.5E-03
447.34	0.49	1.11	1.9E-09	2.5	13.2	2.4	1.8E-01	1.7	8.8E-03
437.32	0.61	1.00	3.0E-03	5.3	12.5	2.6	1.3E-01	2.2	1.2E-01
475.08	1.54	0.93	2.6E-06	2.2	8.5	1.5	2.7E-01	1.4	9.8E-03
465.35	0.58	0.92	1.4E-04	2.3	9.5	1.1	3.0E-02	1.2	3.9E-02
393.29	0.48	0.81	9.2E-10	4.4	13.5	1.3	2.4E-02	2.0	8.8E-03
493.31	0.68	0.66	7.2E-08	1.9	3.9	1.2	3.0E-02	1.2	2.1E-02
467.37	0.63	0.45	2.5E-10	1.9	9.2	1.3	7.4E-02	1.2	2.6E-02
345.27	0.73	0.28	1.3E-08	1.9	5.4	1.2	6.5E-04	1.2	1.5E-02
409.25	0.46	0.17	1.2E-08	3.0	8.0	1.1	2.6E-01	1.3	8.1E-01
536.36	0.58	0.15	1.5E-07	1.8	12.0	1.2	1.9E-01	1.2	8.7E-01

Supplementary Table 1

A list of the features filtered with a %CV <15% along with the associated *p* value, fold change and VIP Score between WT d7 v.s. WT d119.

The fold change and *p* value for the comparison between WT and Tg rats for Day 63 and Day 119 are also included.

		Ctrl (n=5)			PS (n=6)			*p*
BW	(g)	45.8	±	2.08	44.78	±	2.86	0.7806
Glu	(mg/dL)		>600			>600		-
BUN	(mg/dL)	24.6	±	0.86	26.4	±	2.42	0.5887
Cr	(mg/dL)	0.22	±	0.047	0.25	±	0.034	0.6728
AST	(U/L)	132	±	43.0	88.6	±	9.91	0.3108
ALT	(U/L)	74	±	5.76	79.3	±	7.00	0.5814
TG	(mg/dL)	306.6	±	21.8	238.8	±	17.9	0.0384
HDL	(mg/dL)	100.75	±	4.67	100.3	±	4.70	0.9534
LDL	(mg/dL)	642.3	±	130.6	590.6	±	85.7	0.7400

Supplementary Table 2

Clinical data of db/db mouse. The data are expressed as mean ± SEM. Control (Ctrl, n=5) and PS (n=6) data are listed.

BW: body weight; Glu: blood glucose; BUN: blood urine nitrogen; Cr: creatinine; AST: aspartate aminotransferase; ALT: alanine aminotransferase; TG: triglyceride; HDL; high density lipoprotein cholesterol;
LDL: low density lipoprotein cholesterol

		KKAy+HFD Ctrl (n=3)			KKAy+HFD PS (n=4)			*p*
BW	(g)	46.1	±	3.08	47.7	±	2.45	0.7265
Glu	(mg/dL)	321	±	74.1	373	±	61.4	0.6315
Hb	(g/dL)	11.4	±	0.20	12.2	±	0.35	0.1694
Hct	(%)	33.5	±	0.50	36.0	±	1.00	0.1548
BUN	(mg/dL)	15.0	±	7.50	24.0	±	1.00	0.4227
Cr	(mg/dL)	0.3	±	0.00	0.23	±	0.03	0.1161
AST	(U/L)	73.2	±	15.6	82	±	19.8	0.7396
ALT	(U/L)	35.7	±	6.31	35.5	±	5.54	0.9640
TG	(mg/dL)	153	±	23.9	155	±	13.8	0.9504
HDL	(mg/dL)	82.5	±	8.01	87.3	±	9.70	0.8739

Supplementary Table 3

Clinical data from KKAy mice. The data are expressed as mean ± SEM. Control (Ctrl, n=3) and PS (n=4) data are listed.

HFD: high fat diet; BW: body weight; Glu: blood glucose;
BUN: blood urine nitrogen; Cr: creatinine; AST: aspartate aminotransferase;
ALT: alanine aminotransferase; TG: triglyceride;
HDL; high density lipoprotein cholesterol;
LDL: low density lipoprotein cholesterol

	Normo (n=257)	Micro (n=87)	Macro (n=18)	*p*12	*p*13	*p*23
ACR	8.3 (1.0-29.5)	80.1 (30.3-292.6)	516.8 (306.7-6407.4)			
PS	3.1 (0-68.1)	3.6 (0.3-54.1)	6.5 (0.6-26.6)	0.054	0.102	0.634
suPAR	422.5 (142.0-1880.0)	535.6 (168.4-2740.2)	649.1 (257.5-1193.9)	<0.001	0.022	0.634
Age	62.4±12.5	66.1±12.3	63.6±9.2	0.041	0.907	0.721
Gender	150/107	44/43	12/6			
Duration	13.8±8.4	17.2±9.7	13.9±6.8	0.005	0.999	0.306
BMI	24.8±4.1	25.5±5.0	25.6±4.2	0.387	0.777	0.999
SBP	126.4±14.5	130.1±17.0	135.1±12.6	0.121	0.049	0.406
DBP	72.3±9.5	72.2±11.6	76.2±12.4	0.996	0.261	0.284
BS	154.3±56.9	155.2±54.9	148.0±59.1	0.990	0.869	0.851
HbA1c	7.2±1.1	7.1±0.9	7.1±1.2	0.679	0.974	0.979
eGFR	74.7 (31.8-115.4)	71.3 (17.1-109.7)	64.4 (23.2-87.9)	0.009	0.001	0.126
ALT	23.0±12.9	22.0±11.6	18.3±10.6	0.783	0.276	0.499
TC	176.2±31.9	172.3±31.4	179.4±30.5	0.572	0.909	0.656
TG	130.5±83.8	129.4±73.9	169.3±99.7	0.994	0.131	0.149
HDL	56.4±17.2	52.4±14.3	53.5±17.0	0.136	0.757	0.967
UA	5.2±1.8	5.4±1.4	6.1±0.8	0.535	0.083	0.289

Supplementary Table 4

The clinical data for the DKD subjects presented by disease stage in the U-CARE Study.
Mean ± SD was for parametric factors and the Median range was for non-parametric factors (eGFR, ACR, PS and suPAR). Tukey-Kramer test was performed for parametric factor.
Steel-Dwass test was performed for non-parametric factor.
*p*12: Normo vs Micro, *p*13: Normo vs Macro, *p*23: Micro vs Macro.

PS: phenyl sulfate concentration; suPAR: soluble urokinase-type plasminogen activator receptor; Duration: duration of diabetic mellitus; BMI: body mass index; SBP: systolic blood pressure; DBP: diastolic blood pressure; BS: blood glucose level; HbA1c: NGSP value; eGFR: estimated glomerular filtration rate (CKD-EPI); ACR: urinary albumin-to-creatinine ratio;
ALT: alanine aminotransferase; TC: total cholesterol; TG: triglyceride; HDL: high density lipoprotein cholesterol; UA: uremic acid.

	Relation with Log(PS+1)	*p*
Log(PS+1)		
Log suPAR	0.095	0.070
Age	0.216	<0.001
Gender	-0.086	0.104
Duration	0.174	<0.001
BMI	-0.091	0.084
SBP	-0.020	0.708
DBP	-0.057	0.277
BS	-0.017	0.738
HbA1c	-0.111	0.034
Log eGFR	-0.216	<0.001
Log ACR	0.187	<0.001
ALT	-0.004	0.938
TC	0.002	0.968
TG	-0.009	0.870
HDL	0.006	0.902
UA	0.167	0.001

Supplementary Table 5

The correlation between the plasma PS level and various factors was determined based on Spearman Rank-Order Correlation.
The logarithmically converted values of (PS+1), suPAR, eGFR and ACR were used.
In logarithmically conversion of PS, all measured values of PS were added 1.

PS: phenyl sulfate concentration; suPAR: soluble urokinase-type plasminogen activator receptor; Duration: duration of diabetic mellitus; BMI: body mass index; SBP: systolic blood pressure; DBP: diastolic blood pressure; BS: blood glucose concentration; HbA1c: NGSP value;
eGFR: estimated glomerular filtration rate (CKD-EPI); ACR: urinary albumin-to-creatinine ratio; ALT: alanine aminotransferase; TC: total cholesterol level; TG: triglyceride level; HDL: high density lipoprotein cholesterol; UA: uremic acid.

	Normoalbuminuria stage (N=257)					
	Model 1		Model 2		Model 3	
	odds	*p*	odds	*p*	odds	*p*
Log(PS+1)	1.01	0.97	0.88	0.61	0.85	0.56
Log suPAR	2.17	0.10	1.34	0.58	0.94	0.92
Age			1.00	0.995	1.00	0.92
Gender (ref. male)			1.14	0.77	1.23	0.69
BMI			0.96	0.54	0.95	0.46
SBP*			1.03	0.04	1.05	0.01
HbA1c			0.80	0.41	0.78	0.40
Log eGFR*			0.06	0.03	0.08	0.08
Duration					1.03	0.35
DBP					0.99	0.62
ALT					1.02	0.45
TC*					0.98	0.03
TG					1.0002	0.96
HDL*					1.02	0.26
UA					1.09	0.53

	Microalbuminuria stage (N=87)					
	Model 1		Model 2		Model 3	
	odds	*p*	odds	*p*	odds	*p*
Log(PS+1) *	2.09	0.02	2.02	0.04	3.45	0.03
Log suPAR	1.54	0.42	0.97	0.96	2.10	0.42
Age			0.98	0.43	0.97	0.45
Gender (ref. male)			1.22	0.74	1.87	0.44
BMI			0.99	0.86	0.94	0.50
SBP			0.99	0.78	1.04	0.17
HbA1c			1.05	0.90	1.89	0.24
Log eGFR			0.19	0.06	0.31	0.40
Duration					0.91	0.13
DBP					0.95	0.20
ALT*					0.91	0.12
TC					1.01	0.31
TG					0.997	0.68
HDL					1.04	0.24
UA*					3.09	0.005

Supplementary Table 6

Two-year ACR deterioration shown by stratified logistic regression analysis based on the DKD stage. Model 1: only Log(PS+1) and Log suPAR (crude model), Model 2: Model 1 with known factors (age, gender, BMI, SBP, HbA1c and Log eGFR), Model 3: Model 2 with other factors (duration, DBP, ALT, TC, TG, HDL and UA). The 95% confidence interval (95%CI) is listed. Since the number of macroalbuminuria patients was insufficient to analyze, we examined the normoalbuminuria and microalbuminuria groups.

Remaining variables after the stepwise method based on the Akaike's Information Criterion (AIC) in model 3 are depicted as *.

Taqman Gene Expression Assays

Tnfa	Mm00443260_g1
Ccl2	Mm00441242_m1
EMR1	Mm00802529_m1
Tgfb1	Mm01178820_m1
Fn1	Mm01256744_m1
Col1a1	Mm00801666_g1

Supplementary Table 7

List of primers used in the PCR analysis. Primers were purchased from Applied Biosystems. The sequences of the primers and probes are certificated by the company, but not open by company policy.
