# Supplementary material for: Gut microbiome-derived phenyl sulfate contributes to albuminuria in diabetic kidney disease
Source: Nat Commun. 2019 Apr 23;10:1835. doi: 10.1038/s41467-019-09735-4 (PMC6478834; doi:10.1038/s41467-019-09735-4)
Supplement: Supplementary file 2 — Description of Additional Supplementary Files [file 41467_2019_9735_MOESM2_ESM.docx]

**Description of Additional Supplementary Files**

File Name: Supplementary Data 1

Description: Untargeted metabolome analysis of diabetic SLCO4C1-tg rats compared with its littermate.
