# Supplementary material for: Gut microbiome-derived phenyl sulfate contributes to albuminuria in diabetic kidney disease
Source: Nat Commun. 2019 Apr 23;10:1835. doi: 10.1038/s41467-019-09735-4 (PMC6478834; doi:10.1038/s41467-019-09735-4)
Supplement: Supplementary file 4 — Reporting Summary [file 41467_2019_9735_MOESM4_ESM.pdf]

nature research

Corresponding author(s): Takaaki ABE

Last updated by author(s): Mar 26, 2019

Reporting Summary

Nature Research wishes to improve the reproducibility of the work that we publish. This form provides structure for consistency and transparency in reporting. For further information on Nature Research policies, see Authors & Referees and the Editorial Policy Checklist.

Statistics

For all statistical analyses, confirm that the following items are present in the figure legend, table legend, main text, or Methods section.

n/a	Confirmed	
☐	☒	The exact sample size (*n*) for each experimental group/condition, given as a discrete number and unit of measurement
☐	☒	A statement on whether measurements were taken from distinct samples or whether the same sample was measured repeatedly
☐	☒	The statistical test(s) used AND whether they are one- or two-sided<br>*Only common tests should be described solely by name; describe more complex techniques in the Methods section.*
☐	☒	A description of all covariates tested
☐	☒	A description of any assumptions or corrections, such as tests of normality and adjustment for multiple comparisons
☐	☒	A full description of the statistical parameters including central tendency (e.g. means) or other basic estimates (e.g. regression coefficient) AND variation (e.g. standard deviation) or associated estimates of uncertainty (e.g. confidence intervals)
☐	☒	For null hypothesis testing, the test statistic (e.g. *F*, *t*, *r*) with confidence intervals, effect sizes, degrees of freedom and *P* value noted<br>*Give P values as exact values whenever suitable.*
☒	☐	For Bayesian analysis, information on the choice of priors and Markov chain Monte Carlo settings
☒	☐	For hierarchical and complex designs, identification of the appropriate level for tests and full reporting of outcomes
☒	☐	Estimates of effect sizes (e.g. Cohen's *d*, Pearson's *r*), indicating how they were calculated

Our web collection on statistics for biologists contains articles on many of the points above.

Software and code

Policy information about availability of computer code

Data collection	N/A
Data analysis	Microsoft Excel, Microsoft Powerpoint, ImageJ , JUMP software (Pro13, SAS Institute), Adobe Ilustrator CC 2018.

For manuscripts utilizing custom algorithms or software that are central to the research but not yet described in published literature, software must be made available to editors/reviewers. We strongly encourage code deposition in a community repository (e.g. GitHub). See the Nature Research guidelines for submitting code & software for further information.

Data

Policy information about availability of data

All manuscripts must include a data availability statement. This statement should provide the following information, where applicable:

- Accession codes, unique identifiers, or web links for publicly available datasets
- A list of figures that have associated raw data
- A description of any restrictions on data availability

The data that support the findings of this study are available from the corresponding author upon reasonable request.
The source data underlying Figs 1a-b, 1g, 1j, 2a–b, 2d-g, 2i-j, 4b-d, 4f-h, 5a-e and Supplementary Figs 1, 3-7, 9, 16-19, Supplementary Table 2-3 are provided as a Source Data file.
Untargeted metabolome analysis underlying Figs 1c-f, Supplementary Figs 2a-d and Supplementary Table 1 are provided in a file named Supplementary Data 1.
The microbiome data were deposited in the DDBJ database (https://trace.ddbj.nig.ac.jp/DRASearch/) under accession number DRA006253.

Field-specific reporting

Please select the one below that is the best fit for your research. If you are not sure, read the appropriate sections before making your selection.

☒ Life sciences ☐ Behavioural & social sciences ☐ Ecological, evolutionary & environmental sciences

For a reference copy of the document with all sections, see nature.com/documents/nr-reporting-summary-flat.pdf

Life sciences study design

All studies must disclose on these points even when the disclosure is negative.

Sample size	The sample size for each experiment, n, is included in the results section and the associated figure legend.
Data exclusions	No data were excluded from the analyses.
Replication	Data are representatives of at least three independent experiments with similar results and plotted as mean ± SE
Randomization	Mice were random allocated into experimental group
Blinding	The investigators were blinded to group allocation during data collection and analysis.

Reporting for specific materials, systems and methods

We require information from authors about some types of materials, experimental systems and methods used in many studies. Here, indicate whether each material, system or method listed is relevant to your study. If you are not sure if a list item applies to your research, read the appropriate section before selecting a response.

Materials & experimental systems

n/a	Involved in the study	
☐	☒	Antibodies
☐	☒	Eukaryotic cell lines
☒	☐	Palaeontology
☐	☒	Animals and other organisms
☐	☒	Human research participants
☐	☒	Clinical data

Methods

n/a	Involved in the study	
☒	☐	ChIP-seq
☒	☐	Flow cytometry
☒	☐	MRI-based neuroimaging

Antibodies

Antibodies used	The primary antibodies against caludin 1 (abcam#ab15098, 1:1000), occudin (abcam#ab31721, 1:1000) , ZO-1 (Thermo Fisher#40-2200, 1:1000) and GAPDH (abm#G041,1:1000) were purchased from local distributors.
Validation	All primary antibodies were validated by the suppliers.

Eukaryotic cell lines

Policy information about cell lines

Cell line source(s)	SLCO4C1-expressing MDCK (SLCO4C1/MDCKII) cells was established in Tohoku University. Human urinary podocyte cell (HUPEC) were provided by Dr. Jeffrey B. Kopp (NIH, Bethesda). Human kidney proximal tubule cell line HK-2 (CRL2190) and Canine kidney cell line MDCKII (CRL2936) was purchased from ATCC (CRL2936).
Authentication	None of the cell line used were authenticated.
Mycoplasma contamination	None
Commonly misidentified lines (See ICLAC register)	None

Animals and other organisms

Policy information about studies involving animals; ARRIVE guidelines recommended for reporting animal research

Laboratory animals	C57BL/6N Jcl mouse, db/db mouse, KKAy mouse, eNOS Knockout mouse, Akita mouse, SLCO4C1-Tg rat, Wister rat
Wild animals	N/A
Field-collected samples	N/A
Ethics oversight	The animal protocols were approved by the Tohoku University Institutional Animal Care and Use Committee (2016-007-1, 2-16-008-3). This study was conducted in accordance with the Guide for the Care and Use of Laboratory.

Note that full information on the approval of the study protocol must also be provided in the manuscript.

Human research participants

Policy information about studies involving human research participants

Population characteristics	The U-CARE study was a multi-center, observatory clinical study that paned to investigate the urinary biomarkers in diabetic nephropathy (UMIN 00011525). The study design and baseline characteristics were as follows; eligible participants were between 20-80 years of age with type 1 and type 2 diabetes and excluded people with liver cirrhosis and new onset of malignancies within 5 years. Among 777 patients enrolled in 2012, 564 participants were eligible for enrollment in this study. Inclusion criterion was that serum samples stocked in 2014 and clinical data between 2014 and 2016 were available.
Recruitment	described above.
Ethics oversight	For diabetic nephropathy patient cohort study, we recruited 362 patients (U-CARE, registered UMIN 00011525). The analysis was approved by both the Tohoku University Ethics committee (2017-1-870) and Okayama University Ethics committee (1702-026). The collection of human samples was performed in accordance to the Tohoku University (2017-1-870) and after obtaining written informed consent adopted by the Okayama University (UMIN 00011525 and 1702-026).

Note that full information on the approval of the study protocol must also be provided in the manuscript.

Clinical data

Policy information about clinical studies

All manuscripts should comply with the ICMJE guidelines for publication of clinical research and a completed CONSORT checklist must be included with all submissions.

Clinical trial registration	The Urinary biomarker for continuous and rapid progression of diabetic nephropathy (U-CARE) study20 is a multi-center, observatory clinical study that planned to investigate the urinary biomarkers in diabetic nephropathy (UMIN 00011525).
Study protocol	The study design and baseline characteristics were as follows: eligible participants were between 20-80 years of age with type 1 and type 2 diabetes and excluded people with liver cirrhosis and a new onset of malignancies within 5 years.
Data collection	Among 777 patients enrolled in 2012, 564 participants were eligible for enrollment in this study. An included criterion was that serum samples stocked in 2014 and clinical data between 2014 and 2016 would be available. Among the 564 participants, 362 patients with full data were selected.
Outcomes	Parametric factors are presented as means and standard deviations or as medians and interquartile ranges. Non-parametric factors are presented as proportions by the result of Shapiro-Wilk test. As a result, skewed variables (eGFR, ACR, PS and suPAR) were logarithmically transformed to improve normality prior to analysis. Since some PS values in the U-CARE study were calculated as 0 according to the measurement limit, we added 1 to calculate log transformed values. The correlation between the plasma PS level and various factors was calculated using the Spearman Rank-Order Correlation.<br>To perform multiple regression analysis, we built 3 models as an independent variable examined by variance inflation factor (VIF) <10, and the plasma PS level and ACR were used as objective variables. Model 1 is the crude model. Model 2 is adjusted by known factors: age, gender, BMI, SBP, HbA1c, and log (eGFR))23. Model 3 is the full model, adjusted by model 2 plus other clinical factors in Supplementary Table 4 (DBP, ALT, TC, TG, HDL and UA). Since blood sampling did not involve fasting and was strongly related to HbA1c, we did not adjust serum glucose concentration in the following analyses. A logistic regression analysis was also used to identify the factors independently associated with the development of 2-year ACR deterioration. We defined the increased ACR cases as follows63,64: Normoalbuminuric at baseline: a case that progressed from normoalbuminuria to microalbuminuria or macroalbuminuria. Microalbuminuric at baseline: a case that progressed from microalbuminuria to macroalbuminuria or a case with ACR that doubled or more. Macroalbuminuric at baseline: a case with ACR that doubled or more. To identify the factors independently associated with the development of 2-year ACR, the same 3 multiple logistic regression models were used. Model 1: only Log(PS+1) and Log(SuPAR) (crude model). Model 2: Model 1 with known factors (age, gender, BMI, SBP, HbA1c and log(eGFR))23. Model 3: Model 2 with other factors (duration, DBP, ALT, TC, TG, HDL and UA). Results are presented as odds ratios (ORs) with 95% CIs. ORs for all continuous variables were computed for each SD change. *p<0.05 was set as statistically significant. The stepwise method was also performed based on the Akaike's Information Criterion (AIC).
